# Association between time to target mean arterial pressure and 90-day mortality in septic shock: a post hoc analysis of the OPTPRESS trial

**DOI:** 10.1016/j.aicoj.2026.100088

**Published:** 2026-05-14

**Authors:** Ryuto Yokoyama, Tatsuya Hayasaka, Kenya Yarimizu, Kazuma Yamakawa, Takashi Tagami, Yutaka Umemura, Akira Endo

**Affiliations:** a Department of Emergency and Critical Care Medicine, Yamagata University Hospital, Yamagata, Japan; b Department of Anesthesiology, Yamagata University Hospital, Yamagata, Japan; c Department of Emergency and Critical Care Medicine, Osaka Medical and Pharmaceutical University, Osaka, Japan; d Department of Emergency and Disaster Medicine, Jikei University School of Medicine, Tokyo, Japan; e Department of Emergency Medicine, Osaka University Hospital, Osaka, Japan; f Department of Acute Critical Care Medicine, Tsuchiura Kyodo General Hospital, Ibaraki, Japan; g Department of Acute Critical Care and Disaster Medicine, Institute of Science Tokyo, Yushima, Bunkyo, Tokyo, Japan

**Keywords:** Septic shock, Mean arterial pressure, Time to target mean arterial pressure, Vasopressors, Mortality

## Abstract

**Background:**

In septic shock resuscitation, maintaining a mean arterial pressure (MAP) of at least 65 mmHg is recommended; however, the clinical relevance of the time required to achieve target MAP remains unclear. We evaluated the association between time to target MAP (TTT-MAP) and clinical outcomes in septic shock.

**Methods:**

This post hoc secondary analysis used data from the OPTPRESS randomized controlled trial. TTT-MAP was defined as the time from randomization to first attainment of the randomized-arm lower-bound MAP target (80 mmHg in the high-target arm; 65 mmHg in the low-target arm) and categorized as <1 h, 1–3 h, 3–6 h, 6–12 h, ≥12 h, and unreached. The primary outcome was 90-day all-cause mortality. The primary analysis used doubly robust estimation. Secondary analyses included continuous TTT-MAP modeling, Cox proportional hazards models, and assessments of hypotension burden and vasopressor exposure.

**Results:**

Among 516 patients enrolled in OPTPRESS, 476 were included in the complete-case cohort. In the primary six-category analysis, compared with TTT-MAP <1 h, the delayed-attainment category (≥12 h) was associated with higher 90-day mortality (adjusted odds ratio [aOR] 1.48, 95% confidence interval [CI] 1.11–1.98; p = 0.008), whereas 1–3 h, 3–6 h, and 6–12 h were not. In the continuous analysis restricted to patients who achieved target MAP within 24 h, TTT-MAP was not significantly associated with 90-day mortality per 1-h increase (aOR 1.025, 95% CI 0.972–1.082; p = 0.362). In Cox models, prolonged TTT-MAP (≥12 h or unreached) was associated with increased 90-day mortality versus early attainment (<12 h and reached) (adjusted hazard ratio [aHR] 3.24, 95% CI 2.19–4.80; p < 0.001).

**Conclusion:**

In this secondary analysis of the OPTPRESS trial, delayed attainment of the randomized lower-bound MAP target was associated with higher 90-day mortality. However, because TTT-MAP reflects time to first target attainment rather than the hypotension burden itself, these findings should not be interpreted as defining a clinically acceptable duration of hypotension or a threshold effect.

## Highlights

• Longer TTT-MAP was associated with increased 90-day mortality in septic shock.
• TTT-MAP captured the time to first attainment of the assigned MAP target.
• TTT-MAP is distinct from cumulative hypotension burden or recurrent hypotension.
• TTT-MAP may help characterize early hemodynamic restoration processes.

## Background

Sepsis is a major global health issue, with an estimated 49 million cases and ∼11 million deaths annually [1]. Septic shock, characterized by circulatory failure and organ hypoperfusion secondary to severe infection, remains associated with substantial mortality, with in-hospital mortality reaching 30–40% even in contemporary intensive care settings [[2], [3], [4]]. Rapid and appropriate hemodynamic management is therefore essential [4]. Current international guidelines recommend maintaining a mean arterial pressure (MAP) ≥65 mmHg and support early vasopressor initiation with norepinephrine to achieve this target [3,5].

A systematic review and meta-analysis reported that early vasopressor administration may improve survival [6]. Delayed vasopressor initiation has also been associated with increased mortality [7], and norepinephrine initiation within 1 h after shock onset has been linked to improved outcomes [8]. However, more recent evidence has been inconsistent: one meta-analysis found no significant mortality difference between early and delayed norepinephrine administration [9], and a large cohort study reported no association between time from hypotension onset to vasopressor initiation and prognosis [10]. Thus, the optimal timing of vasopressor intervention remains uncertain.

Previous studies in critically ill populations, including patients with septic shock, have reported that hypotension burden, measured using metrics such as time below a MAP threshold, area-under-threshold deficit, and time-weighted average MAP, may be associated with organ dysfunction and mortality [[11], [12], [13], [14]]. However, these associations have varied across studies because of differences in populations, exposure definitions, and analytical approaches.

Although current guidelines recommend target MAP values, they do not specify how quickly these targets should be achieved. Prior studies have reported that early vasopressor strategies can shorten time to target MAP (TTT-MAP) [6,[15], [16], [17]], but direct evidence linking TTT-MAP to 90-day mortality remains limited and inconsistent. Because TTT-MAP does not directly capture hypotension depth, it may reflect a different dimension of resuscitation from conventional hypotension burden metrics. Therefore, we examined the association between TTT-MAP and 90-day mortality in patients with septic shock in a post hoc secondary analysis of the OPTPRESS trial. In this study, TTT-MAP was defined as the time from randomization to first attainment of the assigned lower-bound MAP target.

## Methods

### Study design and data source

This post hoc secondary analysis used individual patient data from the OPTPRESS trial (UMIN000041775). OPTPRESS was a multicenter, open-label, randomized controlled trial conducted at 29 hospitals in Japan in patients aged ≥65 years with septic shock [18]. The study protocol and primary results have been published previously [19]. Patients were randomly assigned in a 1:1 ratio to a high-target or low-target MAP group. The target MAP was 80–85 mmHg in the high-target group and 65–70 mmHg in the low-target group, and these ranges were maintained for 72 h after randomization or until vasopressors were no longer required.

The primary endpoint of the OPTPRESS trial was 90-day all-cause mortality. Secondary endpoints included ventilator-free days, renal replacement therapy-free days, duration of vasopressor therapy, lactate kinetics, and adverse events. The trial found no mortality benefit with the higher MAP target. For the present analysis, prospectively recorded times of randomization and target MAP attainment enabled a time-based definition of the exposure, time to target MAP (TTT-MAP).

This study was reported in accordance with the Strengthening the Reporting of Observational Studies in Epidemiology (STROBE) guidelines (Supplementary Table S1).

### Study population

This secondary analysis used data from the OPTPRESS trial. We first identified patients with valid TTT-MAP ascertainment. TTT-MAP was considered valid when the time from randomization to the first achievement of the assigned lower-bound MAP threshold was available in patients who achieved the target, or when the time variable was missing in patients explicitly recorded as not having achieved the target. Patients with missing or internally inconsistent TTT-MAP ascertainment were excluded.

We then constructed the primary complete-case cohort (n = 476) by excluding patients with missing prespecified covariates required for adjusted analyses. Because baseline lactate was recorded in either mmol/L or mg/dL in the source dataset, lactate values were harmonized before complete-case assessment by preferentially using values recorded in mmol/L and converting mg/dL values to mmol/L when needed. After harmonization, patients with missing values in any prespecified covariate were excluded from the primary analysis. The prespecified covariates were age, sex, body mass index, Sequential Organ Failure Assessment score, initial lactate, initial MAP, corticosteroid use within 72 h, history of hypertension, chronic kidney disease, and malignancy.

### Exposure: TTT-MAP

The primary exposure was TTT-MAP. For the primary categorical analysis, TTT-MAP was classified into six groups: <1 h (reference), 1–3 h, 3–6 h, 6–12 h, ≥12 h, and unreached. This categorization was intended as a pragmatic operational classification of delayed target attainment within this dataset rather than as a biological or clinically actionable threshold. To assess potential dose–response relationships, TTT-MAP was also analyzed as a continuous variable. In the primary categorical analysis, the ≥12 h and unreached categories were prespecified to represent delayed target attainment, informed in part by prior literature on delayed vasopressor intervention [20], while not implying a physiological safety threshold. We also used 6 h as an intermediate cut-point, informed by previous findings indicating that time to peak vasopressor dose in septic shock is approximately 6 h and that peak dose achievement within 6 h is associated with lower mortality [21].

### Definition of TTT-MAP

In the OPTPRESS trial, the target MAP differed by randomized assignment (high-target group, 80–85 mmHg; low-target group, 65–70 mmHg); thus, the target threshold was defined at randomization [18,19]. Accordingly, TTT-MAP was defined as the time from randomization to the first time point at which MAP reached or exceeded the lower bound of the assigned target range (80 mmHg in the high-target group; 65 mmHg in the low-target group). Patients who had received vasopressors for >3 h before randomization were excluded; therefore, the temporal discrepancy between vasopressor initiation and randomization was constrained by the trial eligibility criteria. In the present secondary analysis, TTT-MAP was intended to represent an early attainment-based hemodynamic process variable rather than a direct measure of hypotension burden or tolerable duration of hypotension.

### Maintenance of target MAP in OPTPRESS

In OPTPRESS, hemodynamic management aimed to maintain the assigned target MAP for 72 h after randomization or until vasopressors were no longer required. In contrast, for the present analysis, TTT-MAP was defined as the time to first attainment of the assigned target threshold; sustained maintenance for a prespecified duration was not required. Under the trial protocol, vasopressin could be initiated and titrated up to 0.04 U/min when a norepinephrine dose ≥0.1 μg/kg/min was required to maintain target MAP. In the present analysis, norepinephrine doses were reported in the BASE format, as norepinephrine base-equivalent doses, in accordance with the recent SCCM/ESICM position paper on the reporting of norepinephrine formulations [22]. If target MAP could not be maintained, clinicians could increase norepinephrine and/or add dobutamine or hydrocortisone. Blood pressure was measured either noninvasively using an upper-arm cuff or invasively via an arterial catheter. To improve transparency regarding exposure ascertainment, we additionally summarized the timing and availability of scheduled MAP observations in the available OPTPRESS secondary-analysis datasets. Because these datasets contained MAP values at prespecified nominal study time points rather than raw blood pressure measurement timestamps, these summaries reflect scheduled observation availability and do not fully reconstruct within-window blood pressure measurement frequency.

### Outcome

The primary outcome was 90-day all-cause mortality, consistent with the primary endpoint of the OPTPRESS trial.

### Statistical analysis

Baseline characteristics were summarized as medians (interquartile ranges [IQRs]) for continuous variables and counts (percentages) for categorical variables (Table 1 and Supplementary Table S2).

**Table 1:** Baseline characteristics by time to target mean arterial pressure (TTT-MAP) category.

Variable	Overall (n = 476)	<1 h (n = 91)	1–3 h (n = 173)	3–6 h (n = 93)	6–12 h (n = 65)	≥12 h (n = 36)	Unreached (n = 18)	p value
Age, years	78.00 (73.00–85.00)	77.00 (72.50–85.00)	77.00 (74.00–85.00)	79.00 (74.00–84.00)	80.00 (74.00–84.00)	79.00 (71.00–84.00)	80.50 (77.00–85.00)	0.803
Body mass index, kg/m²	21.40 (18.39–24.54)	21.26 (18.66–23.40)	21.30 (18.07–24.22)	21.68 (18.15–25.33)	22.04 (19.56–24.99)	21.03 (19.00–23.63)	21.60 (17.50–25.94)	0.758
SOFA score	10.00 (8.00–12.00)	10.00 (7.00–12.00)	9.00 (7.00–12.00)	9.00 (8.00–12.00)	10.00 (8.00–12.00)	10.00 (8.00–12.00)	13.00 (10.25–15.00)	0.006
Initial lactate, mmol/L	3.90 (2.67–6.50)	3.83 (2.35–6.11)	3.70 (2.40–6.40)	4.10 (2.80–6.78)	3.40 (2.78–5.53)	4.75 (3.05–6.55)	8.15 (3.65–11.48)	0.024
Initial MAP, mmHg	58.00 (52.00–64.00)	60.00 (53.00–68.00)	58.00 (52.00–64.00)	57.00 (52.00–64.00)	56.00 (52.00–62.00)	57.50 (54.00–64.00)	48.00 (43.00–56.25)	0.002
Male sex	264 (55.5%)	52 (57.1%)	99 (57.2%)	51 (54.8%)	30 (46.2%)	24 (66.7%)	8 (44.4%)	0.377
Corticosteroid use within 72 h	290 (60.9%)	54 (59.3%)	103 (59.5%)	53 (57.0%)	45 (69.2%)	22 (61.1%)	13 (72.2%)	0.595
Hypertension	262 (55.0%)	52 (57.1%)	84 (48.6%)	58 (62.4%)	37 (56.9%)	19 (52.8%)	12 (66.7%)	0.281
Chronic kidney disease	75 (15.8%)	9 (9.9%)	30 (17.3%)	17 (18.3%)	11 (16.9%)	7 (19.4%)	1 (5.6%)	0.418
Malignancy	76 (16.0%)	14 (15.4%)	27 (15.6%)	13 (14.0%)	13 (20.0%)	7 (19.4%)	2 (11.1%)	0.883

#### Primary outcome and doubly robust estimation

To reduce bias from model misspecification, we used doubly robust (DR) estimation, which combines inverse probability of treatment weighting (IPTW) based on propensity scores with outcome regression [23]. This approach remains consistent if either the exposure model or the outcome model is correctly specified and is therefore more robust than methods relying on a single model.

Propensity scores were estimated using multinomial logistic regression treating TTT-MAP as a six-category exposure. The propensity score model included age, sex, body mass index, SOFA score, initial lactate, initial MAP, corticosteroid use within 72 h, history of hypertension, chronic kidney disease, and malignancy. Covariate balance across TTT-MAP categories was assessed using standardized mean differences (SMDs) before and after IPTW. Absolute SMDs <0.10 were considered to indicate good balance; however, residual imbalance, particularly in sparse categories, was considered when interpreting weighted estimates (Supplementary Table S3).

#### Secondary analyses

**Continuous TTT-MAP model:** For analyses treating TTT-MAP as a continuous variable, we included only patients who achieved the assigned lower-bound MAP target within 24 h after randomization; patients who did not reach the target or first reached it after 24 h were excluded.

**Cox proportional hazards models:** We evaluated associations between TTT-MAP and time to death within 90 days using Cox proportional hazards models. Follow-up extended from randomization to day 90. Death within 90 days was treated as the event, and survival at 90 days was censored. Exposure was defined in two ways: (i) prolonged TTT-MAP (≥12 h or unreached) versus early attainment (<12 h and reached), and (ii) five categories: <1 h, 1–3 h, 3–6 h, 6–12 h, and ≥12 h or unreached. Models were adjusted for the same 10 covariates as in the primary analysis, and results were reported as hazard ratios (HRs) with 95% confidence intervals (CIs) and p values. Because TTT-MAP was determined during early treatment, these Cox analyses were used as robustness checks for the primary DR logistic regression. TTT-MAP was not modeled as a time-dependent exposure because it was defined as an early-phase process variable rather than a dynamically updated covariate during follow-up.

**Restricted cubic spline (RCS) analysis:** We assessed potential non-linear associations by modeling continuous TTT-MAP using restricted cubic splines. This analysis was limited to patients who achieved the assigned lower-bound MAP target within 24 h after randomization because TTT-MAP could not be represented on a continuous scale in unreached cases. Relative odds ratios (ORs) were estimated with attainment within 1 h as the reference. This analysis was intended to characterize the adjusted association within the achieved range only.

**Hypotension burden analyses:** Separate from TTT-MAP, we assessed hypotension burden during the first 24 h after vasopressor initiation. We included 442 patients with sufficient systolic and diastolic blood pressure data to calculate MAP over 0–24 h (Supplementary Table S4). Hypotension burden was evaluated at MAP thresholds of 60, 65, 70, 75, and 80 mmHg using two metrics: (i) time below threshold, defined as cumulative hours with MAP below the threshold, with ORs estimated per 1-h increase; and (ii) area-under-threshold (AUC) deficit, defined as the time integral of threshold minus MAP below the threshold (mmHg·h), with ORs estimated per 100 mmHg·h increase. Associations with 90-day mortality were evaluated using multivariable logistic regression adjusted for the same covariates as in the primary analysis.

**Vasopressor escalation and cumulative exposure:** To capture aspects of post-attainment hemodynamic management, we analyzed vasopressor escalation and cumulative exposure (Supplementary Table S5). In the source dataset, norepinephrine exposure was recorded as the cumulative amount of norepinephrine measured in mg for the pre-randomization period and over the first 72 h, together with exposure duration in minutes and body weight in kg. All norepinephrine doses used in these analyses were reported in the BASE format, as norepinephrine base-equivalent doses [22]. When dose normalization was required for sensitivity analyses, these variables were converted to average norepinephrine dose in μg/kg/min. Specifically, we calculated an average pre-randomization escalation-rate proxy (μg/kg/min) using the cumulative norepinephrine dose before randomization (mg), time from vasopressor initiation to randomization (min), and body weight (kg), normalized as cumulative dose/time/body weight. This proxy does not reflect fine-grained titration but approximates the average infusion rate up to randomization. We also evaluated cumulative exposure as (i) cumulative norepinephrine dose before randomization (mg), (ii) cumulative norepinephrine dose over 72 h (mg), and (iii) cumulative vasopressin dose over 72 h (U).

#### Sensitivity analyses

Several prespecified sensitivity analyses were performed to assess the robustness of the primary findings. These included analyses using alternative MAP thresholds, stratification by randomized MAP target group, an alternative covariate set incorporating organ dysfunction indicators, an alternative categorical specification of TTT-MAP, and alternative handling of unreached cases. Detailed definitions and analytic procedures for these sensitivity analyses are provided in Additional file 1: Supplementary Methods (Supplementary Methods S1–S5), and results are demonstrated in Supplementary Tables S6–S10.

For all analyses, statistical significance was set at p < 0.05. All statistical analyses were conducted using Python 3.13 (statsmodels, scikit-learn) and R (version 4.5.2; The R Foundation for Statistical Computing, Vienna, Austria). IPTW was conducted using WeightIt (version 1.5.1), and covariate balance was assessed using cobalt (version 4.6.2).

## Results

### Patient characteristics

**Fig. 1:**
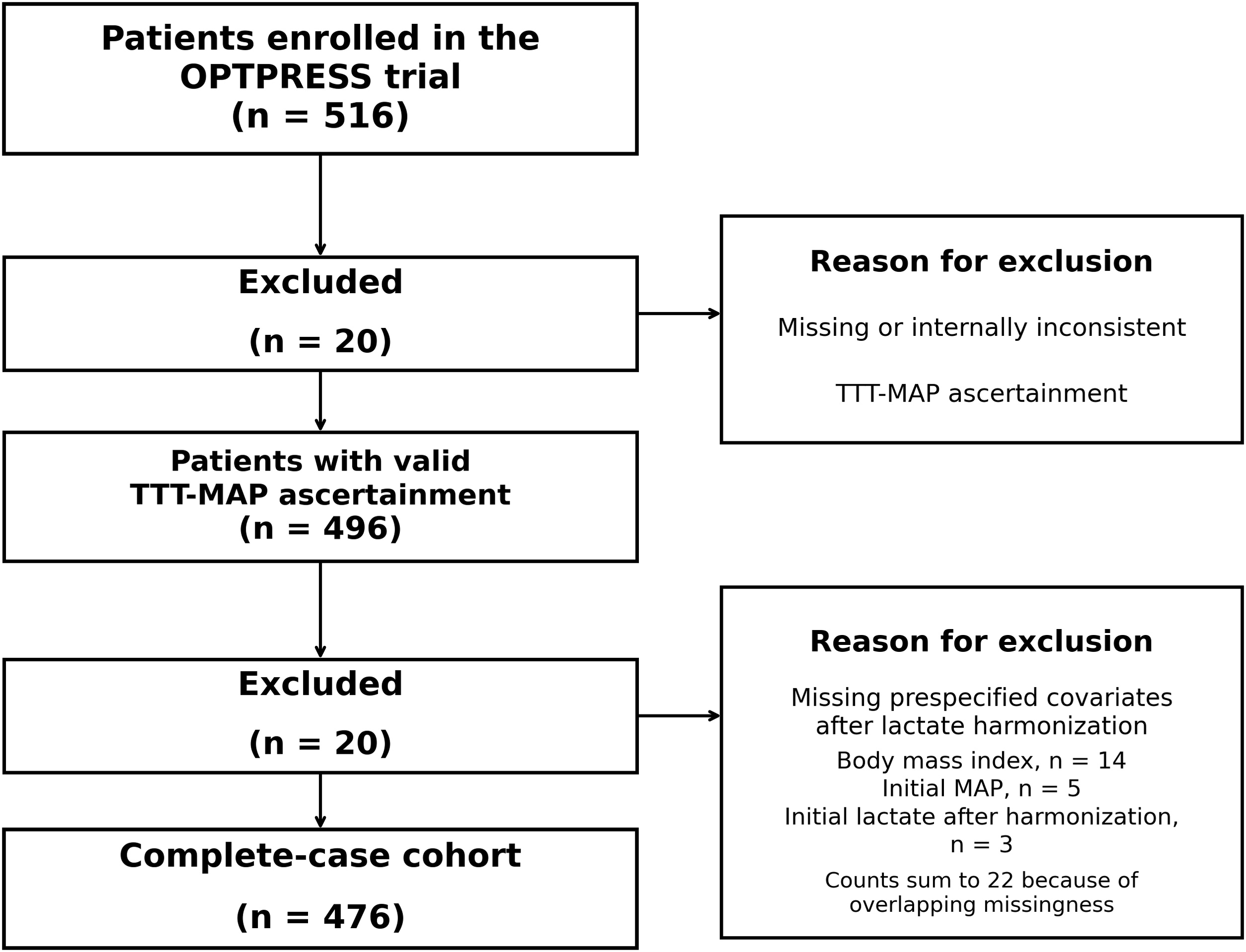
Study flow diagram. Among 516 patients enrolled in the OPTPRESS trial, 20 were excluded because of missing or internally inconsistent TTT-MAP ascertainment. The remaining 496 patients had valid TTT-MAP ascertainment. After harmonization of baseline lactate values recorded in either mmol/L or mg/dL, 20 additional patients were excluded because of missing prespecified covariates, yielding a complete-case cohort of 476 patients. Among these 20 patients, missingness was observed in body mass index (n = 14), initial mean arterial pressure (n = 5), and initial lactate after harmonization (n = 3); these counts sum to 22 because some patients had missingness in more than one covariate. TTT-MAP, time to target mean arterial pressure.

Of the 516 patients enrolled in the OPTPRESS trial, 20 were excluded because of missing or internally inconsistent TTT-MAP ascertainment, leaving 496 patients with valid TTT-MAP ascertainment. After harmonization of baseline lactate values across units, 20 additional patients were excluded because of missing prespecified covariates, resulting in a primary complete-case cohort of 476 patients (Fig. 1). Among these 20 patients, missingness was present for body mass index in 14, initial MAP in 5, and initial lactate after harmonization in 3; these counts exceeded 20 because some patients had missingness in more than one covariate. The median age was 78 years (IQR 73–85), and 55.5% were men (Table 1 and Supplementary Table S2). Table 2 summarizes the primary, secondary, and sensitivity analyses. In addition, to improve transparency regarding exposure ascertainment, supplementary tables summarize the earliest available scheduled MAP observation, the availability of scheduled MAP values at early nominal time points, the mapping between exposure windows and nominal MAP time points available in the datasets, and the availability of at least one scheduled MAP observation within each exposure window by TTT-MAP category (Supplementary Tables S11–S14). In the complete-case cohort, scheduled MAP availability at early nominal time points was high overall, although narrower within-window measurement frequency, particularly for the 1–3 h window, could not be fully reconstructed from the available datasets.

**Table 2:** Primary and key secondary/sensitivity analyses for time to target mean arterial pressure (TTT-MAP) and 90-day mortality association.

Analysis	Population (n)	Exposure definition	Effect estimate (95% CI)	Data location
Primary (DR logistic)	476	TTT-MAP categories (<1 h ref; 1–3; 3–6; 6–12; ≥12; Unreached)	≥12 h vs <1 h: aOR 1.48 (1.11–1.98)	Table 3
Continuous (logistic)	Achieved ≤24 h (n = 448)	Per 1-h increase	aOR 1.025 (0.972–1.082)	Table 4
Cox (binary)	476	Prolonged (≥12 h or unreached) vs early	aHR 3.24 (2.19–4.80)	Table 5
Cox (categorical)	476	≥12 h or unreached vs <1 h	aHR 2.93 (1.77–4.85)	Table 5
RCS	Achieved ≤24 h (n = 448)	Continuous TTT-MAP, reference = 1 h	P for non-linearity = 0.445	Fig. 2
Hypotension burden	442	Time below threshold and AUC deficit	Higher burden at 65–80 mmHg associated with mortality	Supplementary Table S4
Alternative thresholds	442	60, 65, 70, 75, 80 mmHg	Signals observed mainly at 60–70 mmHg	Supplementary Table S6
Sensitivity: organ dysfunction	473	6-category DR + organ dysfunction covariates	TTT-MAP ≥12 h remained associated with higher 90-day mortality; residual imbalance remained after weighting	Supplementary Table S8
Sensitivity: alternative categories	476	1–4 / 4–8 / 8–12 / ≥12 / unreached	Point estimates for ≥12 h were directionally higher but imprecise	Supplementary Table S9
Sensitivity: handling unreached	476	Merged or excluded	Merged analysis remained positive; exclusion analysis was imprecise	Supplementary Table S10

**Legend:** Primary analysis was performed using doubly robust (DR) estimation with inverse probability of treatment weighting (IPTW) based on a multinomial propensity score model, followed by weighted outcome regression. TTT-MAP was defined as the time from randomization to first attainment of the randomized-arm target threshold (low-target group: ≥65 mmHg; high-target group: ≥80 mmHg). The reference category for categorical analyses was TTT-MAP <1 h, unless otherwise specified. “Unreached” indicates failure to attain the target within 24 h and was interpreted descriptively when complete separation occurred; in selected Cox and sensitivity analyses, unreached was combined with ≥12 h to improve estimate stability. Hypotension burden was evaluated over 0–24 h (n = 442) using time below threshold (per 1-h increase) and area-under-threshold deficit (AUC deficit; per 100 mmHg·h increase) across multiple MAP thresholds (60–80 mmHg); detailed results for all thresholds are provided in Supplementary Table S4. Alternative MAP-threshold analyses (Supplementary Table S6) used MAP approximations derived from SBP and DBP recorded at prespecified time points (0, 4, 8, 12, 16, 20, and 24 h). All models were adjusted for the prespecified 10 covariates: age, sex, body mass index, SOFA score, initial lactate, initial MAP, steroid use within 72 h, hypertension, chronic kidney disease, and malignancy. Effect estimates are presented as adjusted odds ratios (aORs) or adjusted hazard ratios (aHRs) with 95% CIs.

Abbreviations: aHR, adjusted hazard ratio; aOR, adjusted odds ratio; AUC, area under the curve; CI, confidence interval; DR, doubly robust; IPTW, inverse probability of treatment weighting; MAP, mean arterial pressure; RCS, restricted cubic spline; SBP, systolic blood pressure; DBP, diastolic blood pressure; SOFA, Sequential Organ Failure Assessment; TTT-MAP, time to target mean arterial pressure.

**Fig. 2:**
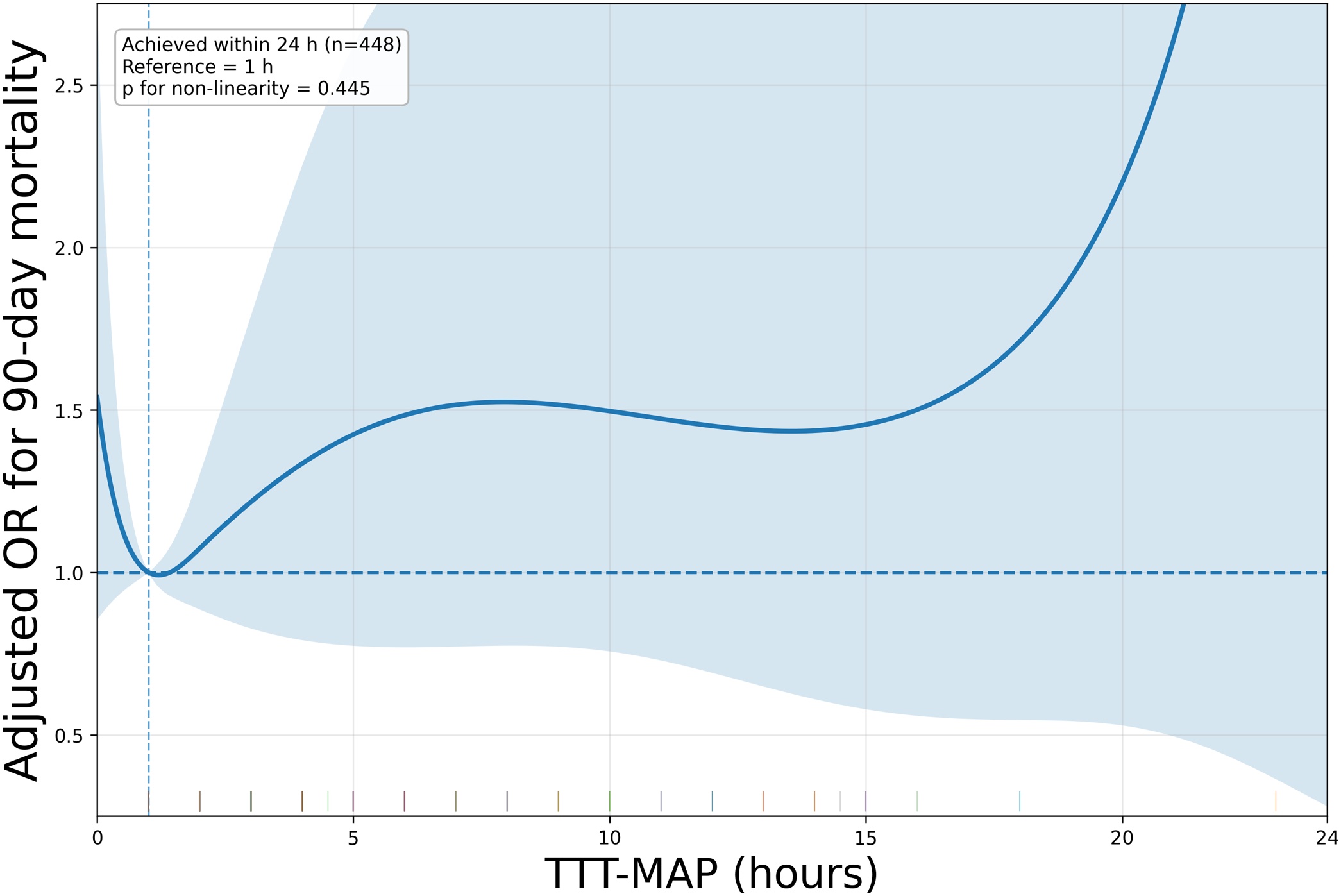
Association between time to target mean arterial pressure and 90-day mortality. Restricted cubic spline analysis of the association between time to target mean arterial pressure (TTT-MAP) and 90-day mortality among patients who achieved the assigned lower-bound MAP target within 24 h after randomization. The curve shows the adjusted odds ratio for 90-day mortality according to continuous TTT-MAP, with 1 h as the reference. Shaded areas indicate 95% confidence intervals. Because patients in the unreached category were excluded, this figure reflects the adjusted association only within the achieved range and should not be interpreted as equivalent to the primary categorical analysis.

### Primary outcome: six-category TTT-MAP analysis

In the DR categorical analysis, TTT-MAP ≥12 h was associated with higher 90-day mortality than that with the reference group (<1 h) (adjusted OR 1.48, 95% CI 1.11–1.98; p = 0.008) (Table 3). No significant differences were observed for 1–3 h, 3–6 h, or 6–12 h. Estimates for the unreached category were interpreted descriptively because of complete separation.

**Table 3:** Doubly robust analysis of TTT-MAP categories and 90-day mortality.

TTT-MAP category	Deaths/n (%)	Crude OR (95% CI)	Adjusted ORα (95% CI)	p value
<1 h (reference)	29 / 91 (31.9)	1.00	1.00	—
1–3 h	45 / 173 (26.0)	0.75 (0.43–1.31)	0.79 (0.58–1.06)	0.112
3–6 h	25 / 93 (26.9)	0.79 (0.42–1.48)	0.81 (0.60–1.08)	0.155
6–12 h	19 / 65 (29.2)	0.88 (0.44–1.77)	1.06 (0.79–1.42)	0.700
≥12 h	16 / 36 (44.4)	1.71 (0.78–3.77)	1.48 (1.11–1.98)	0.008
Unreached	18 / 18 (100.0)	Not estimable owing to complete separation; descriptive only	Not estimable owing to complete separation; descriptive only	－

Crude (unadjusted) and adjusted odds ratios (ORs) for 90-day mortality according to time to target mean arterial pressure (TTT-MAP) category in the primary complete-case cohort (n = 476). Adjusted ORs were estimated using a doubly robust approach combining inverse probability of treatment weighting (IPTW) and weighted multivariable logistic regression. The reference category was TTT-MAP <1 h. Estimates for the unreached category could not be calculated because of complete separation and are therefore presented only descriptively.

Values are presented as odds ratios (ORs) with 95% confidence intervals (CIs).

α: Adjusted using a doubly robust approach combining inverse probability of treatment weighting (IPTW) and multivariable logistic regression. β: Crude OR could not be estimated owing to complete separation.

### Secondary analyses

#### Continuous analysis

In analyses restricted to patients who achieved the assigned lower-bound MAP target within 24 h after randomization (n = 448), continuous TTT-MAP was not significantly associated with 90-day mortality (adjusted OR per 1-h increase 1.025, 95% CI 0.972–1.082; p = 0.362) (Table 4).

**Table 4:** Continuous association between TTT-MAP and 90-day mortality.

Cohort	n	Crude OR (95% CI)	p value	Adjusted OR (95% CI)	p value adj
Overall	448	1.025 (0.974–1.079)	0.335	1.025 (0.972–1.082)	0.362
Low-MAP target	234	1.009 (0.913–1.115)	0.865	1.005 (0.902–1.119)	0.929
High-MAP target	214	1.014 (0.952–1.081)	0.660	1.033 (0.963–1.108)	0.363

Crude (unadjusted) and adjusted odds ratios (ORs) for 90-day mortality per 1 -h increase in time to target mean arterial pressure (TTT-MAP). Continuous analyses included only patients who achieved the assigned lower-bound MAP target within 24 h after randomization; patients who did not reach the target or first reached it after 24 h were excluded (n = 448).

Adjusted models included the following covariates: age, male sex, body mass index, SOFA score, initial lactate, initial MAP, corticosteroid use within 72 h, history of hypertension, chronic kidney disease, and malignancy.

Abbreviations: TTT-MAP, time to target mean arterial pressure; OR, odds ratio; CI, confidence interval; MAP, mean arterial pressure.

#### Cox proportional hazards models

In Cox models (n = 476), prolonged TTT-MAP (≥12 h or unreached) was associated with increased 90-day mortality compared with early attainment (<12 h and reached) (adjusted HR 3.240, 95% CI 2.189–4.796; p < 0.001). In the categorical model, only the ≥12 h or unreached category differed significantly from <1 h (adjusted HR 2.930, 95% CI 1.770–4.849; p < 0.001) (Table 5).

**Table 5:** Sensitivity analyses using Cox proportional hazards models with identical covariates as the primary analysis.

Comparison	Adjusted HR (Cox)	95% CI (lower)	95% CI (upper)	p
Binary exposure model				
Prolonged (TTT-MAP ≥12 h or unreached) vs early (<12 h and reached)	3.240	2.189	4.796	<0.001
Categorical exposure model				
1–3 h	0.840	0.523	1.348	0.469
3–6 h	0.917	0.534	1.574	0.754
6–12 h	0.918	0.510	1.650	0.774
≥12 h or unreached	2.930	1.770	4.849	<0.001

Models were censored at 90 days (Survival Days; event = all-cause mortality within 90 days) and fitted using the Breslow method for ties. All models were adjusted for the same 10 covariates as in the primary analysis: age, sex, body mass index, SOFA score, initial lactate, initial mean arterial pressure, corticosteroid use within 72 h, history of hypertension, chronic kidney disease, and malignancy. The analysis dataset included 476 participants.

#### Spline analysis

In the RCS analysis limited to patients who achieved the assigned lower-bound MAP target within 24 h after randomization (n = 448), no significant evidence of non-linearity (p for non-linearity = 0.445) was noted, and no clear threshold or inflection point was identified. Because unreached cases were excluded, this analysis described the adjusted association within the achieved range rather than directly replicating the primary categorical analysis.

#### Association between hypotension burden and 90-day mortality

Hypotension burden was evaluated in 442 patients with complete MAP derivation over 0–24 h (Supplementary Table S4). Both time-below-threshold and AUC-deficit metrics were associated with 90-day mortality at several thresholds, particularly 65–80 mmHg.

#### Vasopressor escalation and cumulative exposure

The norepinephrine escalation-rate proxy was not associated with 90-day mortality or prolonged TTT-MAP, whereas cumulative 72-h norepinephrine and vasopressin exposure tended to be associated with higher mortality (Supplementary Table S5).

### Sensitivity analyses

#### Association between MAP thresholds and TTT-MAP

In sensitivity analyses redefining TTT using MAP thresholds of 60–80 mmHg, the prolonged category (≥12 h or unreached) was associated with 90-day mortality at thresholds of 60–70 mmHg, with attenuation at higher thresholds (75–80 mmHg) (Supplementary Table S6).

#### Six-category TTT-MAP by randomized MAP group

Exploratory analyses stratified by randomized MAP target group are reported in Supplementary Table S7; estimates were imprecise in sparse categories, and the unreached category remained descriptively poor.

#### Sensitivity analysis incorporating organ dysfunction indicators

In a sensitivity analysis using an alternative covariate set incorporating organ dysfunction–related variables (n = 473), TTT-MAP ≥12 h remained associated with higher 90-day mortality compared with <1 h, whereas the other categories did not show clear differences. Residual imbalance persisted after weighting, supporting cautious interpretation (Supplementary Table S8).

#### Alternative categorization of TTT-MAP

Using alternative category boundaries, point estimates for ≥12 h were directed towards higher mortality but were imprecise (Supplementary Table S9).

#### Sensitivity analyses addressing the handling of unreached cases

Sensitivity analyses addressing the handling of unreached cases are shown in Supplementary Table S10. When the unreached and ≥12 h categories were combined, the prolonged group was associated with 90-day mortality. However, when the unreached cases were excluded, the estimate for ≥12 h was imprecise and not statistically significant.

## Discussion

In this secondary analysis of the OPTPRESS randomized trial dataset, delayed attainment of the randomized lower-bound MAP target was associated with higher 90-day mortality in the primary doubly robust analysis, whereas no clear dose–response pattern was observed within the 1–12 h range. This overall direction was supported by time-to-event analyses, although sensitivity analyses also revealed imprecision when delayed-attainment categories were small. Notably, the ≥12 h category should be interpreted as a pragmatic delayed-attainment category used in this dataset rather than as evidence of a biological or clinically actionable threshold. Accordingly, these findings should not be interpreted as supporting permissive acceptance of delayed resuscitation, as defining a clinically acceptable duration of hypotension, or as demonstrating a threshold effect.

An important interpretive point is that TTT-MAP captures the time to first attainment of the randomized-arm target threshold and does not directly quantify hypotension depth or duration over time, nor recurrent hypotension after attainment. Thus, TTT-MAP is not a substitute for conventional hypotension burden metrics and may reflect a different dimension of early resuscitation, shaped by both clinician-side treatment intensity and patient-level circulatory responsiveness. To address this directly, we evaluated hypotension burden during the first 24 h and found independent associations between time-below-threshold and AUC deficit and 90-day mortality across most thresholds. These findings reproduced, within this dataset, the established prognostic relevance of depth-duration hypotension burden and help contextualize our TTT-MAP results within the broader hypotension literature [[11], [12], [13], [14]].

We also explored vasopressor intensity and exposure as potential correlates of achieving and maintaining target MAP. The norepinephrine escalation-rate proxy was directionally associated with lower mortality but was not statistically significant and was not associated with prolonged TTT-MAP. This suggests that prolonged TTT-MAP cannot be explained simply as “slow vasopressor escalation,” although our proxy may not fully capture bedside titration dynamics because of data granularity. By contrast, higher cumulative 72-h norepinephrine and vasopressin exposure was associated with mortality. These downstream exposure measures likely reflect refractory shock severity and clinician response and may be influenced by confounding by indication and/or mediation; accordingly, they should be interpreted as markers of illness trajectory rather than causal determinants.

To examine whether the findings depended on a single target threshold, we evaluated TTT using alternative MAP thresholds (60–80 mmHg) based on MAP approximations at prespecified 4-h intervals. Delayed attainment (≥12 h/unreached) was associated with mortality at thresholds of 60–70 mmHg, with attenuation at 75–80 mmHg. At higher thresholds, attainment may increasingly reflect physiological recovery, clinician vasopressor intensity, and patient severity, potentially amplifying confounding. In addition, the 4-h sampling interval may have missed rapid attainment in the earliest time window; therefore, threshold-specific findings should be interpreted cautiously.

These findings should also be interpreted in the context of emerging evidence that optimal hemodynamic targets and vasopressor intensity may vary across patients. Permissive hypotension strategies have not shown excess mortality in selected vasodilatory shock populations [24,25], and excessive catecholamine exposure may offset some benefits of rapid shock reversal [[26], [27], [28]]. ANDROMEDA-SHOCK-2 evaluated a multicomponent individualized hemodynamic resuscitation protocol incorporating capillary refill time alongside other perfusion/hemodynamic assessments [29], reinforcing the rationale for integrating process measures with tissue perfusion indices and patient-specific physiology. In this context, TTT-MAP may be best considered an exploratory process variable reflecting treatment intensity, underlying responsiveness, or severity within the broader resuscitation setting, rather than being viewed in isolation to infer an acceptable delay window.

This study has some limitations. First, it was a post hoc secondary analysis of the OPTPRESS trial. Although vasopressor use and target MAP ranges were protocolized, residual confounding cannot be excluded, including from clinician-driven resuscitation, monitoring intensity, and patient-specific circulatory responsiveness. Second, TTT-MAP was defined as time to first attainment of the randomized arm threshold and may have been influenced by measurement frequency and transient threshold crossings; it also does not capture MAP maintenance after attainment or recurrent hypotension. Although the protocol aimed to maintain assigned targets for at least 72 h, post-attainment MAP behavior may still have varied. In addition, in the present secondary analysis, TTT-MAP was based on the recorded time-to-target information available in the OPTPRESS secondary-analysis dataset rather than being newly reconstructed from raw bedside blood pressure measurement timestamps. Third, the association observed in the delayed-attainment category (≥12 h) should not be interpreted as a physiological “safe window,” as categorization and sample distribution may have contributed to imprecision, particularly in delayed-attainment strata. Fourth, all patients who did not reach the target within 24 h died, resulting in complete separation and unstable estimates for the unreached group, which may also have influenced the continuous and spline analyses. Fifth, of the 516 patients enrolled in OPTPRESS, 40 were excluded from the primary analysis because of missing or internally inconsistent TTT-MAP ascertainment or missing prespecified covariates after lactate harmonization, resulting in a complete-case cohort of 476 patients; therefore, selection bias is possible. Sixth, because the analysis was not prospectively powered for between-category comparisons, precision was limited in sparse strata; accordingly, we emphasized uncertainty using 95% CIs rather than post hoc power. Seventh, blood pressure was measured using either non-invasive cuff measurements or invasive arterial pressure monitoring, and measurement density may have varied between and within these monitoring modalities. Additionally, in the available secondary-analysis datasets, MAP values were recorded at prespecified nominal study time points rather than as raw blood pressure measurement timestamps, and arterial-line indicators were not available. Accordingly, although we summarized the availability of scheduled MAP observations in the Supplementary Appendix, we could not reconstruct the exact timing or frequency of actual blood pressure measurements within narrower windows such as 1–3 h. Therefore, apparent time to first target attainment may have been influenced by the underlying hemodynamic trajectory and by monitoring density and modality. Because cuff measurements may also differ from invasive measurements [30,31], exposure misclassification remains a possibility. Eighth, Cox models were included as supportive time-to-event analyses; because TTT-MAP was determined after follow-up began, time-related biases cannot be fully excluded, and these results should be interpreted as associative. Ninth, OPTPRESS enrolled older adults (≥65 years) within a specific protocol and practice setting, which may limit generalizability to younger populations and other care settings. Finally, the onset definition of TTT-MAP is not uniform across studies. In this trial, TTT-MAP was defined as the time from randomization to achievement of the target MAP. This heterogeneity in onset definition remains an important limitation and an issue for future research.

Despite these limitations, we provide a detailed assessment of the association between TTT-MAP and outcomes in septic shock using randomized trial data with explicitly defined target blood pressure ranges and attainment protocols.

## Conclusions

In this secondary analysis of the OPTPRESS trial, delayed attainment of the randomized lower-bound MAP target was associated with higher 90-day mortality. However, because TTT-MAP reflects time to first target attainment rather than the hypotension burden itself, these findings should not be interpreted as defining a clinically acceptable duration of hypotension or a threshold effect. Future prospective studies should be aimed to clarify whether such process-oriented variables, interpreted alongside tissue perfusion indices and patient-specific physiology, provide complementary information for individualized resuscitation strategies.

## Authors’ contributions

R.Y. and A.E. conceptualized the study and curated and visualized the data. R.Y., T.H., and Kenya Y. performed the formal analysis. R.Y. and T.H. developed the methodology. A.E. supervised the work. R.Y. wrote the original draft of the manuscript. Kazuma Y., Y.U., T.T., and A.E. reviewed and edited the manuscript. All authors read and approved the final version of the manuscript.

## Consent for publication

Not applicable.

## Ethics approval and consent to participate

The original OPTPRESS trial was approved by the relevant institutional review boards of the participating institutions (UMIN000041775; ethics approval number: R2020-015). The trial was conducted in accordance with the Declaration of Helsinki, and written informed consent was obtained from all participants in the parent trial. This post hoc secondary analysis using anonymized data from the OPTPRESS trial was approved by the Ethics Committee of Yamagata University Faculty of Medicine (approval No. 2026-10). The requirement for additional informed consent was waived by the committee because this study involved secondary use of anonymized data.

## Declaration of Generative AI and AI-assisted technologies in the writing process

During the preparation of this manuscript, the authors used ChatGPT (OpenAI) solely as an auxiliary tool to assist with language editing and sentence refinement. After using this tool, the authors reviewed and edited the content as needed, and they take full responsibility for the content of the published article.

## Funding

This study was supported by the Japan Society for the Promotion of Science, KAKENHI (grant numbers: 21H03197 and 23K21518). The funding body had no role in the design and conduct of the study; the collection, management, analysis, and interpretation of the data; the preparation, review, or approval of the manuscript; or the decision to submit the manuscript for publication.

## Availability of data and materials

After all ancillary analyses by the trial group, the datasets analyzed in the present study will be available from the corresponding author upon reasonable request and after the approval of the Steering Committee members.

## Declaration of competing interest

Takashi Tagami is an Associate Editor of Annals of Intensive Care but was not involved in the editorial handling or decision-making process for this manuscript. The other authors declare that they have no competing interests.
