# Supplementary material for: Association between time to target mean arterial pressure and 90-day mortality in septic shock: a post hoc analysis of the OPTPRESS trial
Source: Ann Intensive Care. 2026 May 14;16:100088. doi: 10.1016/j.aicoj.2026.100088 (PMC13218114; doi:10.1016/j.aicoj.2026.100088)
Supplement: Supplementary file 1 [file mmc1.docx]

# Additional file 1: Supplementary Appendix

**Title:** Association Between Time to Target Mean Arterial Pressure and 90-Day Mortality in Septic Shock: A Post Hoc Analysis of the OPTPRESS Trial

Ryuto Yokoyama^1^, MD; Tatsuya Hayasaka^2^, MD, PhD; Kenya Yarimizu^2^, MD, PhD; Kazuma Yamakawa^3^, MD, PhD; Takashi Tagami^4^, MD, PhD; Yutaka Umemura^5^, MD, PhD; Akira Endo^6,7^, MD, PhD

1. Department of Emergency and Critical Care Medicine, Yamagata University Hospital, Yamagata, Japan

2. Department of Anesthesiology, Yamagata University Hospital, Yamagata, Japan

3. Department of Emergency and Critical Care Medicine, Osaka Medical and Pharmaceutical University, Osaka, Japan

4. Department of Emergency and Disaster Medicine, Jikei University School of Medicine, Tokyo, Japan

5. Department of Emergency Medicine, Osaka University Hospital, Osaka, Japan

6. Department of Acute Critical Care Medicine, Tsuchiura Kyodo General Hospital, Ibaraki, Japan

7. Department of Acute Critical Care and Disaster Medicine, Institute of Science Tokyo, Yushima, Bunkyo, Tokyo, Japan

**Corresponding author:**

RYUTO YOKOYAMA

Department of Emergency and Critical Care Medicine, Yamagata University Hospital

Address: 2-2-2 Iida Nishi, Yamagata City, Yamagata, Japan

Tel: 023-628-5422

Fax: 023-628-5423

Email: ryusi0311@gmail.com

**Contents:**

Supplementary Table S1. STROBE checklist

Supplementary Methods

Supplementary Method S1. Sensitivity analysis using alternative MAP thresholds

Supplementary Method S2. Stratified analyses by randomized MAP target group

Supplementary Method S3. Sensitivity analysis using organ dysfunction-related covariates

Supplementary Method S4. Sensitivity analysis using alternative TTT-MAP category boundaries

Supplementary Method S5. Sensitivity analysis addressing the handling of unreached cases

Supplementary Table S2. Baseline characteristics by TTT-MAP category (full variable set, n = 476)

Supplementary Table S3. Standardized mean differences before and after inverse probability of treatment weighting across the six TTT-MAP categories

Supplementary Table S4. Depth-duration hypotension metrics (first 24 hours; n = 442)

Supplementary Table S5. Additional analyses of vasopressor escalation rate and total vasopressor exposure (n = 476)

Supplementary Table S6. Sensitivity analysis using alternative MAP thresholds for time-to-threshold and 90-day mortality (n = 442)

Supplementary Table S7. Association between prespecified TTT-MAP categories and 90-day mortality, stratified by randomized MAP target strategy

Supplementary Table S8. Doubly robust sensitivity analysis adjusting for organ dysfunction variables and standardized mean differences

Supplementary Table S9. Association between alternative TTT-MAP categories and 90-day mortality (n = 476)

Supplementary Table S10. Sensitivity analyses for handling the “unreached” group in the doubly robust analysis of TTT-MAP categories and 90-day mortality

Supplementary Table S11. Earliest available scheduled MAP observation in the full OPTPRESS dataset (n=516) and the complete case cohort (n=476)

Supplementary Table S12. Availability of scheduled MAP values at early nominal time points (0–12 h) in the complete case cohort (n=476)

Supplementary Table S13. Mapping between exposure windows and nominal MAP time points available in the datasets

Supplementary Table S14. Availability of at least one scheduled MAP observation within each exposure window, by TTT-MAP category

## Supplementary Table S1. STROBE checklist

|  | Item No. | Recommendation | Page  No. |
| --- | --- | --- | --- |
| **Title and abstract** | 1 | (*a*) Indicate the study’s design with a commonly used term in the title or the abstract | 1 |
|  |  | (*b*) Provide in the abstract an informative and balanced summary of what was done and what was found | 2–4 |
| Introduction | | | |
| Background/rationale | 2 | Explain the scientific background and rationale for the investigation being reported | 4, 5 |
| Objectives | 3 | State specific objectives, including any prespecified hypotheses | 5 |
| Methods | | | |
| Study design | 4 | Present key elements of study design early in the paper | 5, 6 |
| Setting | 5 | Describe the setting, locations, and relevant dates, including periods of recruitment, exposure, follow-up, and data collection | 6 |
| Participants | 6 | Give the eligibility criteria and the sources and methods of selection of participants. Describe methods of follow-up | 6 |
| Variables | 7 | Clearly define all outcomes, exposures, predictors, potential confounders, and effect modifiers. Give diagnostic criteria, if applicable | 6, 7 |
| Data sources/ measurement | 8* | For each variable of interest, give sources of data and details of methods of assessment (measurement). Describe the comparability of assessment methods if there is more than one group | 7–9 |
| Bias | 9 | Describe any efforts to address potential sources of bias | 7, 8 |
| Study size | 10 | Explain how the study size was arrived at | 6, 9 |

| Quantitative variables | 11 | Explain how quantitative variables were handled in the analyses. If applicable, describe which groupings were chosen and why | 6–9 |
| --- | --- | --- | --- |
| Statistical methods | 12 | (a) Describe all statistical methods, including those used to control for confounding | 8, 9 |
|  |  | (b) Describe any methods used to examine subgroups and interactions | 8 |
|  |  | (c) Explain how missing data were addressed | 6, 9 |
|  |  | (d) *Cohort study*—if applicable, explain how loss to follow-up was addressed | NA |
|  |  | (e) Describe any sensitivity analyses | 8,9,10 |
| Participants | 13* | (a) Report numbers of individuals at each stage of the study—e.g., numbers potentially eligible, examined for eligibility, confirmed eligible, included in the study, completed follow-up, and analyzed | 6, 9 |
|  |  | (b) Give reasons for non-participation at each stage | 6 |
|  |  | (c) Consider the use of a flow diagram | Figure 1 |
| Descriptive data | 14* | (a) Give characteristics of study participants (e.g., demographic, clinical, and social) and information on exposures and potential confounders | 6, 7 |
|  |  | (b) Indicate the number of participants with missing data for each variable of interest | 6, 9 |
|  |  | (c) *Cohort study*—Summarize follow-up time (e.g., average and total amount) | NA |
| Outcome data | 15* | *Cohort study*—Report numbers of outcome events or summary measures over time | 7 |

**Results**

| Main results |  | (a) Give unadjusted estimates and, if applicable, confounder-adjusted estimates and their precision (e.g., 95% confidence interval). Make clear which confounders were adjusted for and why they were included | 9, 10 |
| --- | --- | --- | --- |
| Other analyses | 17 | (b) Report category boundaries when continuous variables were categorized | 6, 7 |
| **Discussion** |  |  |  |
| Key results | 18 | (c) If relevant, consider translating estimates of relative risk into absolute risk for a meaningful time period | 10, 11 |
| Limitations | 19 | Discuss limitations of the study, taking into account sources of potential bias or imprecision. Discuss both the direction and magnitude of any potential bias | 13, 14 |
| Interpretation | 20 | Give a cautious overall interpretation of results considering objectives, limitations, multiplicity of analyses, results from similar studies, and other relevant evidence | 12, 13 |
| Generalizability | 21 | Discuss generalizability (external validity) of the study results | 13 |
| Other information | |  |  |
| Funding | 22 | Give the source of funding and the role of the funders for the present study and, if applicable, for the original study on which the present article is based | 16 |

This checklist documents compliance with the STROBE reporting guidelines for observational studies using the OPTPRESS randomized trial dataset. Item numbers correspond to the STROBE statement; the page/line locations indicate where each element is addressed in the revised manuscript and this supplementary appendix.

Supplementary Methods

Supplementary Method S1. Sensitivity analysis using alternative MAP thresholds

To assess whether the primary findings depended on the chosen MAP threshold, we examined the association between time to target mean arterial pressure (TTT-MAP) and 90-day mortality across thresholds of 60, 65, 70, 75, and 80 mmHg. Because the available OPTPRESS secondary-analysis datasets did not contain short-interval continuous MAP recordings, MAP was approximated from systolic and diastolic blood pressure values recorded at prespecified time points (0, 4, 8, 12, 16, 20, and 24 h) using the formula MAP = (SBP + 2 × DBP)/3. For each threshold, TTT-MAP was defined as the first time point at which the approximated MAP reached or exceeded the threshold; patients who did not reach the threshold within 24 h were classified as unreached. TTT-MAP was categorized as <4 h, 4–8 h, 8–12 h, ≥12 h, or unreached, with <4 h set as the reference category. Adjusted odds ratios (ORs) and 95% confidence intervals (CIs) were estimated using multivariable logistic regression with the same prespecified covariates used in the primary analysis. Results are presented in Supplementary Table S6.

Supplementary Method S2. Stratified analyses by randomized MAP target group

To explore whether the association between TTT-MAP and 90-day mortality differed according to randomized MAP target strategy, we performed exploratory stratified analyses in the low-target and high-target groups separately. We first assessed interaction by randomized MAP target group; because the interaction was not statistically significant (p = 0.889), these analyses were interpreted as exploratory rather than as confirmatory. Within each randomized stratum, associations between prespecified TTT-MAP categories and 90-day mortality were evaluated using the same doubly robust framework as in the primary analysis. Because the unreached category exhibited 100% mortality, estimates for this category were presented descriptively. Results are demonstrated in Supplementary Table S7.

Supplementary Method S3. Sensitivity analysis using organ dysfunction-related covariates

To further account for severity using baseline physiologic and organ dysfunction indicators, we performed a sensitivity analysis using the same doubly robust framework employed in the primary analysis but with an alternative covariate set comprising Glasgow Coma Scale, initial mean arterial pressure, PaO2/FiO2 ratio, platelet count, total bilirubin, serum creatinine, age, sex, body mass index, and corticosteroid use within 72 h. Baseline lactate was harmonized before cohort construction, as described in the main manuscript, but was not included in this alternative model. TTT-MAP remained categorized as <1 h, 1–3 h, 3–6 h, 6–12 h, ≥12 h, and unreached. After inverse probability of treatment weighting (IPTW), covariate balance was assessed using standardized mean differences (SMDs), and the maximum absolute SMD for each covariate was calculated across comparisons between each non-reference category and the reference category (<1 h). Results are presented in Supplementary Table S8.

Supplementary Method S4. Sensitivity analysis using alternative TTT-MAP category boundaries

To assess the robustness of the findings to different category boundaries, we reclassified TTT-MAP as <1 h (reference), 1–4 h, 4–8 h, 8–12 h, ≥12 h, and unreached. Associations with 90-day mortality were evaluated using the same doubly robust framework and prespecified covariate set used in the primary analysis. Because the unreached group exhibited complete separation with 100% mortality, estimates for this category were presented descriptively. Results are demonstrated in Supplementary Table S9.

Supplementary Method S5. Sensitivity analysis addressing the handling of unreached cases

Because unreached cases had extreme outcomes and could destabilize estimation, we performed two additional sensitivity analyses focused on the handling of this category. In one analysis, unreached cases were excluded and the analysis was restricted to patients who achieved the target MAP. In the other, unreached cases were combined with the ≥12-h category. Both analyses included adjustment strategies consistent with the primary analysis. Results are presented in Supplementary Table S10.

## Supplementary Table S2. Baseline characteristics by TTT-MAP category (full variable set, n = 476)

| **Variable** | **Overall (n=476)** | **<1 h (n=91)** | **1–3 h (n=173)** | **3–6 h (n=93)** | **6–12 h (n=65)** | **≥12 h (n=36)** | **Unreached (n=18)** | **p-value** |
| --- | --- | --- | --- | --- | --- | --- | --- | --- |
| Age, years | 78.00 (73.00–85.00) | 77.00 (72.50–85.00) | 77.00 (74.00–85.00) | 79.00 (74.00–84.00) | 80.00 (74.00–84.00) | 79.00 (71.00–84.00) | 80.50 (77.00–85.00) | 0.803 |
| Male sex | 264 (55.5%) | 52 (57.1%) | 99 (57.2%) | 51 (54.8%) | 30 (46.2%) | 24 (66.7%) | 8 (44.4%) | 0.377 |
| Body mass index, kg/m² | 21.40 (18.39–24.54) | 21.26 (18.66–23.40) | 21.30 (18.07–24.22) | 21.68 (18.15–25.33) | 22.04 (19.56–24.99) | 21.03 (19.00–23.63) | 21.60 (17.50–25.94) | 0.758 |
| Clinical Frailty Scale before admission | 4.00 (3.00–6.00) | 4.00 (3.00–6.00) | 4.00 (3.00–6.00) | 4.00 (3.00–6.00) | 4.00 (4.00–6.00) | 4.00 (4.00–6.00) | 4.00 (4.00–6.00) | 0.651 |
| Transferred from another hospital | 115 (24.2%) | 21 (23.1%) | 45 (26.0%) | 18 (19.4%) | 19 (29.2%) | 11 (30.6%) | 1 (5.6%) | 0.246 |
| Hypertension | 262 (55.0%) | 52 (57.1%) | 84 (48.6%) | 58 (62.4%) | 37 (56.9%) | 19 (52.8%) | 12 (66.7%) | 0.281 |
| Ischemic heart disease | 51 (10.7%) | 14 (15.4%) | 13 (7.5%) | 6 (6.5%) | 10 (15.4%) | 6 (16.7%) | 2 (11.1%) | 0.130 |
| Chronic heart failure | 87 (18.3%) | 16 (17.6%) | 32 (18.5%) | 21 (22.6%) | 6 (9.2%) | 7 (19.4%) | 5 (27.8%) | 0.319 |
| Chronic obstructive pulmonary disease | 22 (4.6%) | 3 (3.3%) | 9 (5.2%) | 4 (4.3%) | 2 (3.1%) | 1 (2.8%) | 3 (16.7%) | 0.215 |
| Chronic kidney disease | 75 (15.8%) | 9 (9.9%) | 30 (17.3%) | 17 (18.3%) | 11 (16.9%) | 7 (19.4%) | 1 (5.6%) | 0.418 |
| Liver cirrhosis | 11 (2.3%) | 2 (2.2%) | 0 (0.0%) | 4 (4.3%) | 2 (3.1%) | 2 (5.6%) | 1 (5.6%) | 0.135 |
| Diabetes mellitus | 131 (27.5%) | 36 (39.6%) | 46 (26.6%) | 22 (23.7%) | 12 (18.5%) | 10 (27.8%) | 5 (27.8%) | 0.074 |
| Malignancy | 76 (16.0%) | 14 (15.4%) | 27 (15.6%) | 13 (14.0%) | 13 (20.0%) | 7 (19.4%) | 2 (11.1%) | 0.883 |
| Pulmonary focus | 119 (25.0%) | 24 (26.4%) | 42 (24.3%) | 22 (23.7%) | 15 (23.1%) | 12 (33.3%) | 4 (22.2%) | 0.881 |
| Abdominal focus | 147 (30.9%) | 27 (29.7%) | 57 (32.9%) | 33 (35.5%) | 18 (27.7%) | 8 (22.2%) | 4 (22.2%) | 0.618 |
| Urinary tract focus | 128 (26.9%) | 24 (26.4%) | 46 (26.6%) | 19 (20.4%) | 23 (35.4%) | 9 (25.0%) | 7 (38.9%) | 0.330 |
| Soft tissue focus | 44 (9.2%) | 11 (12.1%) | 12 (6.9%) | 12 (12.9%) | 4 (6.2%) | 4 (11.1%) | 1 (5.6%) | 0.461 |
| Bloodstream focus | 17 (3.6%) | 2 (2.2%) | 10 (5.8%) | 2 (2.2%) | 0 (0.0%) | 0 (0.0%) | 3 (16.7%) | 0.006 |
| Initial SBP, mmHg | 79.00 (70.00–90.00) | 82.00 (70.00–95.00) | 79.00 (70.00–87.00) | 80.00 (71.00–90.00) | 76.00 (68.00–88.00) | 80.50 (73.00–91.25) | 73.00 (62.00–81.50) | 0.035 |
| Initial DBP, mmHg | 46.00 (40.00–53.00) | 49.00 (42.00–55.50) | 47.00 (41.00–53.00) | 46.00 (40.00–53.00) | 45.00 (40.00–52.00) | 47.50 (42.50–53.25) | 38.00 (34.25–42.75) | 0.007 |
| Initial MAP, mmHg | 58.00 (52.00–64.00) | 60.00 (53.00–68.00) | 58.00 (52.00–64.00) | 57.00 (52.00–64.00) | 56.00 (52.00–62.00) | 57.50 (54.00–64.00) | 48.00 (43.00–56.25) | 0.002 |
| Initial heart rate, bpm | 102.00 (87.00–118.00) | 104.00 (88.50–121.50) | 102.00 (87.00–118.00) | 99.00 (89.00–117.00) | 102.00 (86.00–114.00) | 103.50 (86.50–117.25) | 107.50 (70.25–125.50) | 0.970 |
| Initial pH | 7.39 (7.30–7.45) | 7.38 (7.32–7.45) | 7.39 (7.30–7.45) | 7.39 (7.30–7.44) | 7.39 (7.33–7.45) | 7.40 (7.30–7.46) | 7.24 (7.14–7.31) | 0.005 |
| Initial PaCO2, mmHg | 32.90 (27.40–39.70) | 33.20 (27.35–39.35) | 33.70 (27.00–41.00) | 32.30 (27.40–38.80) | 32.70 (27.90–38.20) | 30.25 (28.50–36.62) | 35.10 (23.50–42.20) | 0.871 |
| Initial PaO2, mmHg | 98.65 (78.00–146.25) | 98.15 (78.50–133.50) | 99.00 (80.00–158.00) | 99.50 (77.97–128.50) | 101.00 (78.80–142.00) | 92.30 (66.80–143.50) | 92.20 (66.40–211.00) | 0.968 |
| Initial bicarbonate, mmol/L | 19.70 (15.95–22.80) | 20.40 (17.10–22.80) | 19.90 (15.90–23.20) | 20.00 (15.90–22.50) | 20.20 (16.40–23.10) | 19.40 (17.15–22.65) | 13.20 (11.30–17.30) | 0.011 |
| Initial FiO2 | 40.00 (30.00–90.00) | 40.00 (29.00–85.00) | 45.00 (30.00–90.00) | 40.00 (29.50–90.00) | 40.00 (28.00–75.00) | 45.00 (30.00–80.00) | 95.00 (45.00–100.00) | 0.237 |
| Initial lactate, mmol/L | 3.90 (2.67–6.50) | 3.83 (2.35–6.11) | 3.70 (2.40–6.40) | 4.10 (2.80–6.78) | 3.40 (2.78–5.53) | 4.75 (3.05–6.55) | 8.15 (3.65–11.48) | 0.024 |
| Fluid until randomization, mL | 1500.00 (1000.00–2148.12) | 1704.00 (1035.00–2475.00) | 1500.00 (1000.00–2262.50) | 1500.00 (847.50–2000.00) | 1500.00 (1000.00–2000.00) | 1285.00 (982.50–2102.50) | 1225.00 (1000.00–2062.50) | 0.368 |
| Norepinephrine dose until randomization, mg | 0.40 (0.10–1.00) | 0.50 (0.11–0.90) | 0.30 (0.02–0.92) | 0.54 (0.10–1.20) | 0.36 (0.10–1.20) | 0.55 (0.15–1.21) | 0.57 (0.21–1.91) | 0.235 |
| Duration from vasopressor initiation to randomization, min | 60.00 (20.00–116.00) | 75.00 (30.00–120.00) | 60.00 (12.50–114.00) | 60.00 (15.75–120.00) | 50.00 (15.00–90.00) | 59.00 (24.75–86.25) | 89.50 (39.75–116.25) | 0.378 |
| SOFA score | 10.00 (8.00–12.00) | 10.00 (7.00–12.00) | 9.00 (7.00–12.00) | 9.00 (8.00–12.00) | 10.00 (8.00–12.00) | 10.00 (8.00–12.00) | 13.00 (10.25–15.00) | 0.006 |
| P/F ratio | 250.00 (154.50–368.50) | 240.00 (148.50–354.00) | 256.00 (157.00–385.00) | 269.00 (155.75–385.50) | 243.00 (167.00–357.00) | 220.50 (162.75–313.00) | 188.50 (81.25–257.75) | 0.101 |
| Platelet count | 140.00 (81.50–229.00) | 139.50 (87.50–243.50) | 142.00 (97.00–223.00) | 141.00 (80.00–246.00) | 138.00 (81.00–232.00) | 156.50 (68.25–232.00) | 108.50 (43.50–178.50) | 0.527 |
| Bilirubin, mg/dL | 0.90 (0.60–1.40) | 0.90 (0.60–1.58) | 0.90 (0.60–1.40) | 0.90 (0.60–1.36) | 0.90 (0.60–1.50) | 0.97 (0.60–1.60) | 1.10 (0.75–1.57) | 0.748 |
| GCS | 13.00 (9.00–14.00) | 13.00 (10.00–14.00) | 13.00 (7.00–14.00) | 13.00 (9.00–15.00) | 14.00 (10.00–15.00) | 13.00 (9.00–14.00) | 11.00 (6.00–14.00) | 0.253 |
| Creatinine, mg/dL | 1.72 (1.15–2.80) | 1.61 (0.96–2.54) | 1.66 (1.01–2.76) | 1.63 (1.19–2.60) | 2.00 (1.30–3.02) | 2.28 (1.19–3.54) | 2.63 (1.83–3.51) | 0.078 |
| Corticosteroid use within 72 h | 290 (60.9%) | 54 (59.3%) | 103 (59.5%) | 53 (57.0%) | 45 (69.2%) | 22 (61.1%) | 13 (72.2%) | 0.595 |
| Arrhythmia adverse event | 47 (9.9%) | 5 (5.5%) | 20 (11.6%) | 8 (8.6%) | 7 (10.8%) | 7 (19.4%) | 0 (0.0%) | 0.135 |

Continuous variables are presented as median (interquartile range) and categorical variables as n (%). P-values were calculated using the Kruskal–Wallis test for continuous variables and the chi-square test for categorical variables. For multi-level categorical variables, a single p-value is shown for the first level.

This table presents an expanded set of baseline characteristics across the six prespecified TTT-MAP categories to complement Table 1 in the main manuscript. Variables include demographics, comorbidities, physiological and laboratory indices at enrollment, and early treatments. Summary statistics and p-values are intended for descriptive purposes; no multiplicity adjustment was applied.

## Supplementary Table S3. Standardized mean differences before and after inverse probability of treatment weighting across the six TTT-MAP categories

| **Covariate** | Comparison | **SMD_unweighted** | **SMD_IPTW-weighted** | **Balanced (<0.10) after IPTW** |
| --- | --- | --- | --- | --- |
| Age (years) | 1–3 h vs <1 h (reference) | 0.022 | -0.055 | Yes |
| Age (years) | 3–6 h vs <1 h (reference) | 0.098 | -0.068 | Yes |
| Age (years) | 6–12 h vs <1 h (reference) | 0.061 | -0.049 | Yes |
| Age (years) | ≥12 h vs <1 h (reference) | -0.050 | -0.028 | Yes |
| Age (years) | Unreached vs <1 h (reference) | 0.309 | -0.084 | Yes |
| Male sex | 1–3 h vs <1 h (reference) | 0.002 | 0.014 | Yes |
| Male sex | 3–6 h vs <1 h (reference) | -0.046 | 0.041 | Yes |
| Male sex | 6–12 h vs <1 h (reference) | -0.221 | -0.035 | Yes |
| Male sex | ≥12 h vs <1 h (reference) | 0.197 | 0.048 | Yes |
| Male sex | Unreached vs <1 h (reference) | -0.256 | 0.161 | No |
| Body mass index | 1–3 h vs <1 h (reference) | 0.016 | 0.003 | Yes |
| Body mass index | 3–6 h vs <1 h (reference) | 0.161 | -0.008 | Yes |
| Body mass index | 6–12 h vs <1 h (reference) | 0.227 | 0.037 | Yes |
| Body mass index | ≥12 h vs <1 h (reference) | 0.096 | 0.017 | Yes |
| Body mass index | Unreached vs <1 h (reference) | 0.295 | 0.317 | No |
| SOFA score | 1–3 h vs <1 h (reference) | -0.038 | -0.021 | Yes |
| SOFA score | 3–6 h vs <1 h (reference) | -0.086 | -0.039 | Yes |
| SOFA score | 6–12 h vs <1 h (reference) | 0.038 | -0.019 | Yes |
| SOFA score | ≥12 h vs <1 h (reference) | 0.279 | 0.024 | Yes |
| SOFA score | Unreached vs <1 h (reference) | 0.965 | 0.280 | No |
| Baseline lactate | 1–3 h vs <1 h (reference) | 0.039 | 0.007 | Yes |
| Baseline lactate | 3–6 h vs <1 h (reference) | 0.233 | 0.010 | Yes |
| Baseline lactate | 6–12 h vs <1 h (reference) | -0.019 | -0.030 | Yes |
| Baseline lactate | ≥12 h vs <1 h (reference) | 0.285 | -0.025 | Yes |
| Baseline lactate | Unreached vs <1 h (reference) | 0.783 | 0.184 | No |
| Baseline MAP | 1–3 h vs <1 h (reference) | -0.268 | 0.013 | Yes |
| Baseline MAP | 3–6 h vs <1 h (reference) | -0.225 | 0.032 | Yes |
| Baseline MAP | 6–12 h vs <1 h (reference) | -0.400 | -0.022 | Yes |
| Baseline MAP | ≥12 h vs <1 h (reference) | -0.044 | 0.075 | Yes |
| Baseline MAP | Unreached vs <1 h (reference) | -1.126 | -0.118 | No |
| Corticosteroid within 72 h | 1–3 h vs <1 h (reference) | 0.004 | -0.028 | Yes |
| Corticosteroid within 72 h | 3–6 h vs <1 h (reference) | -0.048 | -0.054 | Yes |
| Corticosteroid within 72 h | 6–12 h vs <1 h (reference) | 0.208 | -0.052 | Yes |
| Corticosteroid within 72 h | ≥12 h vs <1 h (reference) | 0.036 | -0.181 | No |
| Corticosteroid within 72 h | Unreached vs <1 h (reference) | 0.274 | 0.801 | No |
| History of hypertension | 1–3 h vs <1 h (reference) | -0.173 | -0.065 | Yes |
| History of hypertension | 3–6 h vs <1 h (reference) | 0.107 | -0.067 | Yes |
| History of hypertension | 6–12 h vs <1 h (reference) | -0.004 | -0.103 | No |
| History of hypertension | ≥12 h vs <1 h (reference) | -0.088 | -0.022 | Yes |
| History of hypertension | Unreached vs <1 h (reference) | 0.197 | 0.263 | No |
| Chronic kidney disease | 1–3 h vs <1 h (reference) | 0.219 | -0.008 | Yes |
| Chronic kidney disease | 3–6 h vs <1 h (reference) | 0.243 | -0.026 | Yes |
| Chronic kidney disease | 6–12 h vs <1 h (reference) | 0.208 | 0.011 | Yes |
| Chronic kidney disease | ≥12 h vs <1 h (reference) | 0.273 | -0.068 | Yes |
| Chronic kidney disease | Unreached vs <1 h (reference) | -0.163 | -0.274 | No |
| Malignancy | 1–3 h vs <1 h (reference) | 0.006 | -0.035 | Yes |
| Malignancy | 3–6 h vs <1 h (reference) | -0.040 | -0.024 | Yes |
| Malignancy | 6–12 h vs <1 h (reference) | 0.121 | -0.042 | Yes |
| Malignancy | ≥12 h vs <1 h (reference) | 0.107 | -0.017 | Yes |
| Malignancy | Unreached vs <1 h (reference) | -0.126 | 0.443 | No |

This table reports standardized mean differences (SMDs) before and after inverse probability of treatment weighting (IPTW) for the six-category TTT-MAP exposure. For this multi-category exposure, balance was assessed by comparing each non-reference category with the reference category (<1 h [reference]). IPTW was estimated using a multinomial logistic model with the same prespecified covariates used in both the weighting and outcome models of the primary doubly robust analysis. Absolute |SMD| values <0.10 were considered indicative of adequate balance. After weighting, balance improved for several covariates, but residual imbalance remained, particularly in comparisons involving the Unreached category; this informed cautious interpretation.

Abbreviations: SMD, standardized mean difference; IPTW, inverse probability of treatment weighting; TTT-MAP, time-to-target mean arterial pressure.

## Supplementary Table S4. Depth-duration hypotension metrics (first 24 hours; n = 442)

**Association between hypotension burden (0–24 h) and 90-day mortality across thresholds**

| **Threshold (mmHg)** | **Metric** | **N** | **Deaths** | **Crude OR (95% CI)** | **Crude p value** | **Adjusted OR (95% CI)** | **Adjusted p-value** |
| --- | --- | --- | --- | --- | --- | --- | --- |
| 60 | Time below (per 1 h) | 442 | 120 | 1.07 (1.02–1.13) | 0.010 | 1.07 (1.01–1.13) | 0.027 |
| 60 | AUC deficit (per 100 mmHg·h) | 442 | 120 | 1.87 (1.06–3.28) | 0.030 | 1.53 (0.82–2.88) | 0.184 |
| 65 | Time below (per 1 h) | 442 | 120 | 1.07 (1.03–1.11) | <0.001 | 1.07 (1.03–1.11) | <0.001 |
| 65 | AUC deficit (per 100 mmHg·h) | 442 | 120 | 1.70 (1.17–2.46) | 0.006 | 1.58 (1.04–2.40) | 0.034 |
| 70 | Time below (per 1 h) | 442 | 120 | 1.04 (1.01–1.07) | 0.004 | 1.05 (1.02–1.09) | 0.001 |
| 70 | AUC deficit (per 100 mmHg·h) | 442 | 120 | 1.54 (1.19–1.99) | 0.001 | 1.53 (1.15–2.05) | 0.004 |
| 75 | Time below (per 1 h) | 442 | 120 | 1.03 (1.01–1.06) | 0.010 | 1.05 (1.02–1.08) | 0.002 |
| 75 | AUC deficit (per 100 mmHg·h) | 442 | 120 | 1.36 (1.13–1.63) | 0.001 | 1.39 (1.13–1.70) | 0.002 |
| 80 | Time below (per 1 h) | 442 | 120 | 1.03 (1.00–1.06) | 0.038 | 1.04 (1.01–1.07) | 0.006 |
| 80 | AUC deficit (per 100 mmHg·h) | 442 | 120 | 1.26 (1.09–1.45) | 0.002 | 1.30 (1.11–1.52) | 0.001 |

We quantified the hypotension burden using (i) cumulative time below MAP thresholds and (ii) area-under-threshold deficit (AUC deficit; mmHg·h), computed from serial MAP measurements (at 0, 4, 8, 12, and 24 h) using linear interpolation. Logistic regression models report crude and adjusted ORs with robust (HC1) standard errors; adjusted models include age, male sex, BMI, SOFA, lactate, initial MAP, steroid within 72 h, hypertension, CKD, and malignancy.

## Supplementary Table S5. Additional analyses of vasopressor escalation rate and total vasopressor exposure (n=476)

| **Exposure** | **Variant** | **Unit** | **N (90-d death model)** | **aOR for 90-d death** | **95% CI (death)** | **p-value (death)** |
| --- | --- | --- | --- | --- | --- | --- |
| NA escalation rate (µg/kg/min) | Raw | per 0.1 | 402 | 0.998 | 0.987–1.009 | 0.746 |
| NA escalation rate (µg/kg/min) | Winsorized (cap at 99th=4.26) | per 0.1 | 402 | 0.955 | 0.909–1.004 | 0.073 |
| Total NA dose until randomization (mg) | Raw | per 1 | 475 | 1.063 | 0.957–1.181 | 0.254 |
| Total NA dose until randomization (mg) | Winsorized (cap at 99th=5.00) | per 1 | 475 | 1.000 | 0.815–1.228 | 0.997 |
| Total NA dose over 72 h (mg) | Raw | per 10 | 473 | 1.166 | 1.055–1.289 | 0.003 |
| Total NA dose over 72 h (mg) | Winsorized (cap at 99th=110.26) | per 10 | 473 | 1.178 | 1.061–1.309 | 0.002 |
| Total vasopressin dose over 72 h (U) | Raw | per 10 | 473 | 1.066 | 1.017–1.118 | 0.008 |
| Total vasopressin dose over 72 h (U) | Winsorized (cap at 99th=170.05) | per 10 | 473 | 1.071 | 1.019–1.126 | 0.006 |

We performed multivariable logistic regression adjusted for baseline covariates (randomization arm, age, sex, body mass index, SOFA score, initial lactate, initial MAP, corticosteroid use within 72 h, and comorbidities [hypertension, chronic kidney disease, malignancy]). Associations are presented as adjusted odds ratios (ORs) with 95% confidence intervals per prespecified unit. Winsorized analyses capped the exposure at the 99th percentile to reduce the influence of extreme values. TTT-MAP prolongation was defined as ≥12 h or target not achieved.

Panel A. Association with 90-day all-cause mortality

Panel B. Association with prolonged TTT-MAP (≥12 h or unreached)

| **Exposure** | **Variant** | **Unit** | **N (TTT prolong model)** | **aOR for TTT prolong** | **95% CI (TTT)** | **p value (TTT)** |
| --- | --- | --- | --- | --- | --- | --- |
| NA escalation rate (µg/kg/min) | Raw | per 0.1 | 402 | 0.993 | 0.962–1.024 | 0.635 |
| NA escalation rate (µg/kg/min) | Winsorized (cap at 99th=4.26) | per 0.1 | 402 | 0.999 | 0.948–1.052 | 0.960 |
| Total NA dose until randomization (mg) | Raw | per 1 | 475 | 1.014 | 0.830–1.238 | 0.894 |
| Total NA dose until randomization (mg) | Winsorized (cap at 99th=5.00) | per 1 | 475 | 1.091 | 0.815–1.462 | 0.557 |
| Total NA dose over 72 h (mg) | Raw | per 10 | 473 | 1.271 | 1.119–1.443 | <0.001 |
| Total NA dose over 72 h (mg) | Winsorized (cap at 99th=110.26) | per 10 | 473 | 1.305 | 1.139–1.496 | <0.001 |
| Total vasopressin dose over 72 h (U) | Raw | per 10 | 473 | 1.013 | 0.954–1.075 | 0.677 |
| Total vasopressin dose over 72 h (U) | Winsorized (cap at 99th=170.05) | per 10 | 473 | 1.016 | 0.948–1.089 | 0.652 |

## Supplementary Table S6. Sensitivity analysis using alternative MAP thresholds for time-to-threshold and 90-day mortality (n = 442)

Panel A. Sample size and crude 90-day mortality by threshold and time-to-threshold category

| **MAP threshold (mmHg)** | **Time-to-threshold category** | **N** | **Crude 90-day mortality (%)** |
| --- | --- | --- | --- |
| 60 | <4 h | 281 | 26.3 |
| 60 | 4–8 h | 133 | 26.3 |
| 60 | 8–12 h | 23 | 34.8 |
| 60 | ≥12 h or unreached | 5 | 60.0 |
| 65 | <4 h | 194 | 23.2 |
| 65 | 4–8 h | 183 | 27.9 |
| 65 | 8–12 h | 39 | 33.3 |
| 65 | ≥12 h or unreached | 26 | 42.3 |
| 70 | <4 h | 138 | 21.7 |
| 70 | 4–8 h | 174 | 24.1 |
| 70 | 8–12 h | 74 | 35.1 |
| 70 | ≥12 h or unreached | 56 | 39.3 |
| 75 | <4 h | 98 | 25.5 |
| 75 | 4–8 h | 140 | 22.1 |
| 75 | 8–12 h | 83 | 30.1 |
| 75 | ≥12 h or unreached | 121 | 32.2 |
| 80 | <4 h | 67 | 25.4 |
| 80 | 4–8 h | 112 | 21.4 |
| 80 | 8–12 h | 77 | 24.7 |
| 80 | ≥12 h or unreached | 186 | 32.3 |

We assessed whether the association between time-to-target mean arterial pressure (TTT-MAP) and 90-day all-cause mortality was sensitive to specific MAP thresholds. Because continuous MAP recordings were not available for this sensitivity analysis, MAP at prespecified time points (0, 4, 8, 12, 16, 20, and 24 h after enrollment) was approximated from systolic and diastolic blood pressure as MAP = (SBP + 2×DBP)/3. For each candidate threshold (60, 65, 70, 75, and 80 mmHg), time-to-threshold was defined as the earliest time point at which the approximated MAP met or exceeded the threshold; patients who did not reach the threshold by 24 h were classified as “unreached.” For regression analyses, time-to-threshold was categorized as <4 h, 4–8 h, 8–12 h, and ≥12 h or unreached, with <4 h as the reference. Adjusted odds ratios (aORs) and 95% confidence intervals (CIs) were estimated using multivariable logistic regression adjusted for a fixed set of 10 baseline covariates: age, sex, body mass index, SOFA score, initial lactate, initial MAP, corticosteroid use within 72 h, and comorbidities (hypertension, chronic kidney disease, and malignancy).

Panel B. Adjusted association with 90-day mortality (multivariable logistic regression; 10 covariates)

| **MAP threshold (mmHg)** | **Category (vs <4 h)** | **Adjusted OR** | **95% CI** | **p-value** |
| --- | --- | --- | --- | --- |
| 60 | 4–8 h | 0.87 | 0.51–1.49 | 0.618 |
| 60 | 8–12 h | 1.09 | 0.41–2.93 | 0.865 |
| 60 | ≥12 h or unreached | 2.71 | 0.35–21.02 | 0.339 |
| 65 | 4–8 h | 1.18 | 0.70–1.97 | 0.539 |
| 65 | 8–12 h | 1.51 | 0.68–3.37 | 0.311 |
| 65 | ≥12 h or unreached | 2.33 | 0.91–5.95 | 0.076 |
| 70 | 4–8 h | 1.12 | 0.62–2.01 | 0.705 |
| 70 | 8–12 h | 2.02 | 1.02–4.00 | 0.043 |
| 70 | ≥12 h or unreached | 2.31 | 1.10–4.87 | 0.027 |
| 75 | 4–8 h | 0.83 | 0.43–1.59 | 0.571 |
| 75 | 8–12 h | 1.37 | 0.68–2.77 | 0.377 |
| 75 | ≥12 h or unreached | 1.49 | 0.78–2.84 | 0.230 |
| 80 | 4–8 h | 0.79 | 0.37–1.70 | 0.551 |
| 80 | 8–12 h | 1.22 | 0.55–2.73 | 0.628 |
| 80 | ≥12 h or unreached | 1.64 | 0.83–3.24 | 0.157 |

Supplementary Table S7. Association between prespecified TTT-MAP categories and 90-day mortality, stratified by randomized MAP target strategy

**Low-target group**

| **TTT-MAP category** | **Deaths / n (%)** | **Crude OR (95% CI)** | **Adjusted OR (95% CI)** | **p-value** |
| --- | --- | --- | --- | --- |
| <1 h (reference) | 20/72 (27.8) | 1.00 | 1.00 | - |
| 1–3 h | 23/100 (23.0) | 0.78 (0.39–1.56) | 0.98 (0.57–1.66) | 0.928 |
| 3–6 h | 9/35 (25.7) | 0.90 (0.36–2.25) | 0.75 (0.45–1.24) | 0.260 |
| 6–12 h | 5/21 (23.8) | 0.81 (0.26–2.51) | 0.38 (0.22–0.67) | <0.001 |
| ≥12 h | 2/6 (33.3) | 1.30 (0.22–7.66) | 0.99 (0.58–1.68) | 0.976 |
| Unreached | 3/3 (100.0) | Not estimable | Not estimable | - |

This table shows the association between prespecified TTT-MAP categories and 90-day mortality after stratification by randomized MAP target strategy (low-target vs. high-target). Crude odds ratios (ORs) were estimated using univariable logistic regression models including only TTT-MAP categories. Adjusted ORs were estimated using a doubly robust approach that combined inverse probability of treatment weighting based on a multinomial propensity score model with outcome regression adjusted for the same pre-specified covariates as in the primary analysis. These stratified analyses were exploratory and were performed to assess directional consistency across randomized MAP target strategies rather than to make definitive claims regarding effect heterogeneity.

**High-target group**

| **TTT-MAP category** | **Deaths / n (%)** | **Crude OR (95% CI)** | **Adjusted OR (95% CI)** | **p-value** |
| --- | --- | --- | --- | --- |
| <1 h (reference) | 9/19 (47.4) | 1.00 | 1.00 | - |
| 1–3 h | 22/73 (30.1) | 0.48 (0.17–1.34) | 0.32 (0.21–0.49) | <0.001 |
| 3–6 h | 16/58 (27.6) | 0.42 (0.15–1.23) | 0.33 (0.23–0.49) | <0.001 |
| 6–12 h | 14/44 (31.8) | 0.52 (0.17–1.56) | 0.48 (0.31–0.73) | <0.001 |
| ≥12 h | 14/30 (46.7) | 0.97 (0.31–3.07) | 0.86 (0.58–1.27) | 0.455 |
| Unreached | 15/15 (100.0) | Not estimable | Not estimable | - |

Abbreviations: TTT-MAP, time to target mean arterial pressure; MAP, mean arterial pressure; OR, odds ratio; CI, confidence interval.

Notes:
(i) The reference category was <1 h within each randomized MAP target stratum.
(ii) The covariates used in the adjusted models were the same as those used in the primary analysis.
(iii) The Unreached category exhibited complete separation because all patients died within this category; therefore, crude and adjusted ORs were not reliably estimable and are not reported.
(iv) Because some subgroup counts were small, particularly in the low-target group, these exploratory estimates should be interpreted cautiously.

## Supplementary Table S8. Doubly robust sensitivity analysis adjusting for organ dysfunction variables and standardized mean differences

| **TTT-MAP category (reference: <1 h)** | **Deaths/Total (%)** | **Crude OR (95% CI)** | **Adjusted OR (95% CI)** | **p-value** |
| --- | --- | --- | --- | --- |
| <1h | 28/89 (31.5) | 1.00 | 1.00 |  |
| 1–3 h | 45/173 (26.0) | 0.77 (0.44–1.34) | 0.75 (0.56–1.01) | 0.059 |
| 3–6 h | 25/92 (27.2) | 0.81 (0.43–1.54) | 0.93 (0.69–1.25) | 0.625 |
| 6–12 h | 19/65 (29.2) | 0.90 (0.45–1.81) | 0.87 (0.64–1.18) | 0.366 |
| ≥12 h | 16/36 (44.4) | 1.74 (0.79–3.86) | 1.57 (1.18–2.08) | 0.002 |
| Unreached | 18/18 (100.0) | Not estimable due to complete separation; descriptive only | Not estimable due to complete separation; descriptive only | — |

| **Covariate** | Comparison | **SMD_unweighted** | **SMD_IPTW-weighted** | **Balanced (<0.10) after IPTW** |
| --- | --- | --- | --- | --- |
| Age (years) | 1–3 h vs <1 h (reference) | 0.024 | -0.071 | Yes |
| Age (years) | 3–6 h vs <1 h (reference) | 0.118 | -0.065 | Yes |
| Age (years) | 6–12 h vs <1 h (reference) | 0.063 | -0.155 | No |
| Age (years) | ≥12 h vs <1 h (reference) | -0.048 | -0.052 | Yes |
| Age (years) | Unreached vs <1 h (reference) | 0.31 | -0.111 | No |
| Male sex | 1–3 h vs <1 h (reference) | 0.021 | 0.006 | Yes |
| Male sex | 3–6 h vs <1 h (reference) | -0.037 | 0.015 | Yes |
| Male sex | 6–12 h vs <1 h (reference) | -0.202 | -0.03 | Yes |
| Male sex | ≥12 h vs <1 h (reference) | 0.217 | 0.122 | No |
| Male sex | Unreached vs <1 h (reference) | -0.236 | 0.267 | No |
| Body mass index | 1–3 h vs <1 h (reference) | 0.014 | -0.016 | Yes |
| Body mass index | 3–6 h vs <1 h (reference) | 0.17 | -0.04 | Yes |
| Body mass index | 6–12 h vs <1 h (reference) | 0.223 | 0.03 | Yes |
| Body mass index | ≥12 h vs <1 h (reference) | 0.093 | -0.046 | Yes |
| Body mass index | Unreached vs <1 h (reference) | 0.292 | 0.397 | No |
| Corticosteroids within 72 h | 1–3 h vs <1 h (reference) | 0.0 | -0.002 | Yes |
| Corticosteroids within 72 h | 3–6 h vs <1 h (reference) | -0.039 | -0.037 | Yes |
| Corticosteroids within 72 h | 6–12 h vs <1 h (reference) | 0.203 | -0.053 | Yes |
| Corticosteroids within 72 h | ≥12 h vs <1 h (reference) | 0.032 | -0.174 | No |
| Corticosteroids within 72 h | Unreached vs <1 h (reference) | 0.27 | 0.52 | No |
| Glasgow Coma Scale | 1–3 h vs <1 h (reference) | -0.18 | 0.006 | Yes |
| Glasgow Coma Scale | 3–6 h vs <1 h (reference) | -0.025 | 0.005 | Yes |
| Glasgow Coma Scale | 6–12 h vs <1 h (reference) | 0.123 | -0.024 | Yes |
| Glasgow Coma Scale | ≥12 h vs <1 h (reference) | -0.124 | -0.029 | Yes |
| Glasgow Coma Scale | Unreached vs <1 h (reference) | -0.443 | 0.011 | Yes |
| Initial MAP (mmHg) | 1–3 h vs <1 h (reference) | -0.299 | 0.012 | Yes |
| Initial MAP (mmHg) | 3–6 h vs <1 h (reference) | -0.25 | -0.011 | Yes |
| Initial MAP (mmHg) | 6–12 h vs <1 h (reference) | -0.434 | -0.01 | Yes |
| Initial MAP (mmHg) | ≥12 h vs <1 h (reference) | -0.073 | 0.026 | Yes |
| Initial MAP (mmHg) | Unreached vs <1 h (reference) | -1.164 | -0.066 | Yes |
| P/F ratio | 1–3 h vs <1 h (reference) | 0.132 | 0.023 | Yes |
| P/F ratio | 3–6 h vs <1 h (reference) | 0.15 | 0.016 | Yes |
| P/F ratio | 6–12 h vs <1 h (reference) | 0.108 | -0.037 | Yes |
| P/F ratio | ≥12 h vs <1 h (reference) | 0.001 | 0.003 | Yes |
| P/F ratio | Unreached vs <1 h (reference) | -0.69 | -0.74 | No |
| Platelet count | 1–3 h vs <1 h (reference) | -0.008 | 0.012 | Yes |
| Platelet count | 3–6 h vs <1 h (reference) | 0.004 | 0.006 | Yes |
| Platelet count | 6–12 h vs <1 h (reference) | 0.013 | -0.033 | Yes |
| Platelet count | ≥12 h vs <1 h (reference) | -0.041 | -0.057 | Yes |
| Platelet count | Unreached vs <1 h (reference) | -0.612 | -0.35 | No |
| Total bilirubin | 1–3 h vs <1 h (reference) | -0.224 | 0.029 | Yes |
| Total bilirubin | 3–6 h vs <1 h (reference) | -0.209 | -0.036 | Yes |
| Total bilirubin | 6–12 h vs <1 h (reference) | -0.003 | -0.018 | Yes |
| Total bilirubin | ≥12 h vs <1 h (reference) | -0.055 | 0.11 | No |
| Total bilirubin | Unreached vs <1 h (reference) | -0.241 | 0.064 | Yes |
| Serum creatinine | 1–3 h vs <1 h (reference) | 0.205 | 0.085 | Yes |
| Serum creatinine | 3–6 h vs <1 h (reference) | 0.01 | -0.007 | Yes |
| Serum creatinine | 6–12 h vs <1 h (reference) | 0.337 | 0.015 | Yes |
| Serum creatinine | ≥12 h vs <1 h (reference) | 0.483 | 0.07 | Yes |
| Serum creatinine | Unreached vs <1 h (reference) | 0.605 | -0.095 | Yes |

## This table presents a sensitivity analysis using the same doubly robust framework as in the primary analysis, but with an alternative covariate set reflecting organ dysfunction–related variables. Complete-case analysis was performed in 473 patients. The propensity score and weighted outcome models included age, male sex, body mass index, corticosteroid use within 72 h, Glasgow Coma Scale, initial mean arterial pressure, PaO2/FiO2 ratio, platelet count, total bilirubin, and serum creatinine (10 covariates in total). TTT-MAP was categorized as <1 h, 1–3 h, 3–6 h, 6–12 h, ≥12 h, and unreached, with the unreached group retained as a separate category. Doubly robust estimates are shown together with standardized mean differences (SMDs) before and after inverse probability of treatment weighting (IPTW). Covariate balance was assessed by comparing each non-reference category with the reference category (<1 h [reference]); absolute |SMD| values <0.10 were considered indicative of adequate balance. Estimates for the unreached category were interpreted descriptively because of complete separation.

## Supplementary Table S9. Association between alternative TTT-MAP categories and 90-day mortality (n = 476)

| **TTT-MAP category** | **N** | **90-day mortality** | **Crude OR (95% CI)** | **p-value** | **DR adjusted OR (95% CI)** | **p-value adj** |
| --- | --- | --- | --- | --- | --- | --- |
| <1 h (reference) | 91 | 29/91 (31.9%) | 1.00 (ref) | — | 1.00 (ref) | — |
| 1–4 h | 221 | 56/221 (25.3%) | 0.73 (0.42–1.24) | 0.240 | 0.77 (0.57–1.03) | 0.081 |
| 4–8 h | 78 | 25/78 (32.1%) | 1.01 (0.53–1.93) | 0.980 | 1.19 (0.89–1.59) | 0.233 |
| 8–12 h | 32 | 8/32 (25.0%) | 0.71 (0.29–1.78) | 0.467 | 0.79 (0.59–1.06) | 0.119 |
| ≥12 h | 36 | 16/36 (44.4%) | 1.71 (0.78–3.77) | 0.184 | 1.53 (1.15–2.04) | 0.004 |
| Unreached | 18 | 18/18 (100.0%) | Not estimable due to complete separation; descriptive only | － | Not estimable due to complete separation; descriptive only | － |

This analysis assesses robustness to alternative category boundaries for TTT-MAP (<1 h, 1–4 h, 4–8 h, 8–12 h, ≥12 h, and unreached). Adjusted ORs are estimated using the same doubly robust framework and covariate set as those of the primary analysis. As delayed-attainment categories may be small and the unreached group exhibits complete separation, estimates are interpreted with attention to confidence interval width and stability.

DR-adjusted ORs were estimated using a doubly robust approach (IPTW based on multinomial propensity scores + outcome regression). Models adjusted for age, sex, BMI, SOFA score, baseline lactate, baseline MAP, steroid use within 72 h, history of hypertension, chronic kidney disease, and malignancy (same covariate set as the primary analysis). The “unreached” category had 100% mortality, causing complete separation; estimates for this category should be interpreted descriptively.

## Supplementary Table S10. Sensitivity analyses for handling the “unreached” group in the doubly robust analysis of TTT-MAP categories and 90-day mortality

| **TTT-MAP category** | **Deaths / n (%)** | **Crude OR (95% CI)** | **Adjusted OR± (95% CI)** | **p-value** |
| --- | --- | --- | --- | --- |
|  | | | | |
| <1 h (reference) | 29/91 (31.9) | 1.00 | 1.00 | — |
| 1–3 h | 45/173 (26.0) | 0.75 (0.43–1.31) | 0.79 (0.59–1.06) | 0.119 |
| 3–6 h | 25/93 (26.9) | 0.79 (0.42–1.48) | 0.82 (0.61–1.10) | 0.176 |
| 6–12 h | 19/65 (29.2) | 0.88 (0.44–1.77) | 1.06 (0.79–1.41) | 0.708 |
| ≥12 h or unreached | 34/54 (63.0) | 3.63 (1.79–7.37) | 2.75 (2.07–3.65) | <0.001 |
|  | | | | |
| <1 h (reference) | 29/91 (31.9) | 1.00 | 1.00 | — |
| 1–3 h | 45/173 (26.0) | 0.75 (0.43–1.31) | 0.75 (0.55–1.02) | 0.065 |
| 3–6 h | 25/93 (26.9) | 0.79 (0.42–1.48) | 0.76 (0.56–1.03) | 0.075 |
| 6–12 h | 19/65 (29.2) | 0.88 (0.44–1.77) | 1.06 (0.78–1.43) | 0.712 |
| ≥12 h | 16/36 (44.4) | 1.71 (0.78–3.77) | 1.42 (1.06–1.91) | 0.019 |

These sensitivity analyses address the handling of unreached cases, which have extreme outcomes and can destabilize estimation. Two approaches are reported: (i) excluding unreached cases and restricting to those who achieved target MAP, and (ii) combining unreached with the ≥12-h category. Data are presented as crude and doubly robust adjusted ORs using the same covariate set as the primary analysis, to evaluate whether conclusions depend on a single analytic choice.

Values are presented as odds ratios (ORs) with 95% confidence intervals (CIs). Crude ORs were calculated from unweighted 2×2 contingency tables using the Wald method. ±Adjusted ORs were estimated using a doubly robust approach combining inverse probability of treatment weighting (IPTW) and multivariable logistic regression, consistent with the main analysis.

In the main analysis, the unreached group showed complete separation (90-day mortality 18/18), making the crude OR not estimable when modeled as a separate category; therefore, we evaluated alternative strategies by merging the unreached group with ≥12 h category and by excluding the unreached group.

Supplementary Table S11. Earliest available scheduled MAP observation in the full OPTPRESS dataset (n=516) and the complete case cohort (n=476)

*Full OPTPRESS dataset (n = 516).*

| **First available scheduled MAP** | **n** | **%** |
| --- | --- | --- |
| 0 h | 499 | 96.7% |
| No scheduled MAP available | 15 | 2.9% |
| 4 h | 1 | 0.2% |
| 8 h | 1 | 0.2% |

Complete case cohort (n=476)

| **First available scheduled MAP** | **n** | **%** |
| --- | --- | --- |
| 0 h | 475 | 99.8% |
| 4 h | 1 | 0.2% |

This table summarizes the earliest available scheduled MAP observation in the full OPTPRESS dataset (n=516) and in the complete case cohort (n=476). Because the available secondary-analysis datasets contained MAP values at prespecified nominal study time points rather than raw blood pressure measurement timestamps, these summaries reflect scheduled observation availability rather than actual measurement timing.

Abbreviations: MAP, mean arterial pressure; OPTPRESS, Optimal target blood pressure in elderly with septic shock trial.

Supplementary Table S12. Availability of scheduled MAP values at early nominal time points (0–12 h) in the complete case cohort (n=476)

*Overall* complete case cohort (n=476)

| **Group** | **N** | **0 h** | **4 h** | **8 h** | **12 h** |
| --- | --- | --- | --- | --- | --- |
| Complete case cohort | 476 | 475/476 (99.8%) | 472/476 (99.2%) | 467/476 (98.1%) | 458/476 (96.2%) |

*By TTT-MAP exposure group in the* complete case cohort (n=476)

| **TTT-MAP exposure group** | **N** | **0 h** | **4 h** | **8 h** | **12 h** |
| --- | --- | --- | --- | --- | --- |
| <1 h (reference) | 91 | 91/91 (100.0%) | 90/91 (98.9%) | 89/91 (97.8%) | 89/91 (97.8%) |
| 1–3 h | 173 | 172/173 (99.4%) | 172/173 (99.4%) | 173/173 (100.0%) | 170/173 (98.3%) |
| 3–6 h | 93 | 93/93 (100.0%) | 93/93 (100.0%) | 92/93 (98.9%) | 92/93 (98.9%) |
| 6–12 h | 65 | 65/65 (100.0%) | 65/65 (100.0%) | 65/65 (100.0%) | 65/65 (100.0%) |
| ≥12 h | 36 | 36/36 (100.0%) | 36/36 (100.0%) | 36/36 (100.0%) | 36/36 (100.0%) |
| Unreached | 18 | 18/18 (100.0%) | 16/18 (88.9%) | 12/18 (66.7%) | 6/18 (33.3%) |

This table summarizes the availability of scheduled MAP values at early nominal time points (0–12 h) in the complete case cohort (n=476) overall and by TTT-MAP exposure group. Percentages denote the proportion of patients with a non-missing scheduled MAP value at each nominal time point. These summaries are based on scheduled observation availability in the secondary-analysis datasets and do not represent actual bedside measurement frequency.

Abbreviations: MAP, mean arterial pressure; TTT-MAP, time to target mean arterial pressure.

Supplementary Table S13. Mapping between exposure windows and nominal MAP time points available in the datasets

| **Exposure window requested by reviewer** | **Nominal MAP time points available in the uploaded datasets** | **Patients with ≥1 scheduled MAP in window** | **Patients with all scheduled MAPs present in window** |
| --- | --- | --- | --- |
| <1 h | 0 h | 475/476 (99.8%) | 475/476 (99.8%) |
| 1–3 h | Not available | Not reconstructable | Not reconstructable |
| 3–6 h | 4 h | 472/476 (99.2%) | 472/476 (99.2%) |
| 6–12 h | 8 h, 12 h | 467/476 (98.1%) | 458/476 (96.2%) |
| 12–24 h | 16 h, 20 h, 24 h | 456/476 (95.8%) | 444/476 (93.3%) |
| 24–72 h | 28 h, 32 h, 36 h, 40 h, 44 h, 48 h, 52 h, 56 h, 60 h, 64 h, 68 h, 72 h | 442/476 (92.9%) | 371/476 (77.9%) |

This table maps the exposure windows to the nominal MAP time points available in the OPTPRESS secondary-analysis datasets. Because the available datasets did not contain raw blood pressure measurement timestamps, arterial-line indicators, or device-level measurement-frequency data, within-window measurement counts could not be fully reconstructed, particularly for the 1–3 h window.

Abbreviations: MAP, mean arterial pressure; OPTPRESS, Optimal target blood pressure in elderly with septic shock trial.

Supplementary Table S14. Availability of at least one scheduled MAP observation within each exposure window, by TTT-MAP category

| **TTT-MAP exposure group** | **N** | **<1 h** | **1–3 h** | **3–6 h** | **6–12 h** | **12–24 h** | **24–72 h** |
| --- | --- | --- | --- | --- | --- | --- | --- |
| <1 h (reference) | 91 | 91/91 (100.0%) | NR | 90/91 (98.9%) | 89/91 (97.8%) | 89/91 (97.8%) | 88/91 (96.7%) |
| 1–3 h | 173 | 172/173 (99.4%) | NR | 172/173 (99.4%) | 173/173 (100.0%) | 171/173 (98.8%) | 165/173 (95.4%) |
| 3–6 h | 93 | 93/93 (100.0%) | NR | 93/93 (100.0%) | 92/93 (98.9%) | 91/93 (97.8%) | 88/93 (94.6%) |
| 6–12 h | 65 | 65/65 (100.0%) | NR | 65/65 (100.0%) | 65/65 (100.0%) | 64/65 (98.5%) | 61/65 (93.8%) |
| ≥12 h | 36 | 36/36 (100.0%) | NR | 36/36 (100.0%) | 36/36 (100.0%) | 36/36 (100.0%) | 36/36 (100.0%) |
| Unreached | 18 | 18/18 (100.0%) | NR | 16/18 (88.9%) | 12/18 (66.7%) | 5/18 (27.8%) | 4/18 (22.2%) |

This table reports, for each TTT-MAP exposure group, the proportion of patients with at least one scheduled MAP observation available within each exposure window among the nominal time points represented in the datasets. “NR” indicates windows that were not reconstructable from the available datasets. These summaries reflect scheduled observation availability rather than actual within-window measurement frequency.

Abbreviations: MAP, mean arterial pressure; NR, not reconstructable from available data; TTT-MAP, time to target mean arterial pressure.
